# Adaptation of Oxidative Phosphorylation Machinery Compensates for Hepatic Lipotoxicity in Early Stages of MAFLD

**DOI:** 10.3390/ijms23126873

**Published:** 2022-06-20

**Authors:** Pia Fahlbusch, Aleksandra Nikolic, Sonja Hartwig, Sylvia Jacob, Ulrike Kettel, Cornelia Köllmer, Hadi Al-Hasani, Stefan Lehr, Dirk Müller-Wieland, Birgit Knebel, Jörg Kotzka

**Affiliations:** 1 Institute of Clinical Biochemistry and Pathobiochemistry, German Diabetes Center at the Heinrich-Heine-University Duesseldorf, Leibniz Center for Diabetes Research, 40225 Duesseldorf, Germany; pia.fahlbusch@ddz.de (P.F.); aleksandra.nikolic@ddz.de (A.N.); sonja.hartwig@ddz.de (S.H.); sylvia.jacob@ddz.de (S.J.); ulrike.kettel@ddz.de (U.K.); cornelia.koellmer@ddz.de (C.K.); hadi.al-hasani@ddz.de (H.A.-H.); stefan.lehr@ddz.de (S.L.); joerg.kotzka@ddz.de (J.K.); 2 German Center for Diabetes Research (DZD), Partner Duesseldorf, 40225 Duesseldorf, Germany; 3 Medical Faculty, Heinrich-Heine-University Düsseldorf, 40225 Düsseldorf, Germany; 4 Clinical Research Centre, Department of Internal Medicine I, University Hospital Aachen, 52074 Aachen, Germany; dirmueller@ukaachen.de

**Keywords:** fatty liver, hepatic metabolism, mitochondria, substrate, subunit

## Abstract

Alterations in mitochondrial function are an important control variable in the progression of metabolic dysfunction-associated fatty liver disease (MAFLD), while also noted by increased de novo lipogenesis (DNL) and hepatic insulin resistance. We hypothesized that the organization and function of a mitochondrial electron transport chain (ETC) in this pathologic condition is a consequence of shifted substrate availability. We addressed this question using a transgenic mouse model with increased hepatic insulin resistance and DNL due to constitutively active human SREBP-1c. The abundance of ETC complex subunits and components of key metabolic pathways are regulated in the liver of these animals. Further omics approaches combined with functional assays in isolated liver mitochondria and primary hepatocytes revealed that the SREBP-1c-forced fatty liver induced a substrate limitation for oxidative phosphorylation, inducing enhanced complex II activity. The observed increased expression of mitochondrial genes may have indicated a counteraction. In conclusion, a shift of available substrates directed toward activated DNL results in increased electron flows, mainly through complex II, to compensate for the increased energy demand of the cell. The reorganization of key compounds in energy metabolism observed in the SREBP-1c animal model might explain the initial increase in mitochondrial function observed in the early stages of human MAFLD.

## 1. Introduction

Mitochondrial activity provides the energy for key metabolic pathways in the liver, notably the synthesis and breakdown of fatty acids and glucose. Consequently, liver mitochondria contribute to anaplerosis as well as cataplerosis and therefore represent an important control mechanism for hepatic and systemic metabolism, dependent on substrate availability. In this study, we investigated a mouse model with simple hepatic steatosis to elucidate the molecular mechanisms behind the mitochondrial respiratory dynamics in early-stage fatty liver disease. Mice are physiologically well-characterized [1,2] and resemble an early-stage fatty liver phenotype, without hepatocellular damage, inflammation, or fibrosis [1,2,3], due to transgenic liver-specific overexpression of the human sterol regulatory element-binding protein (SREBP)-1c [2]. SREBP-1c is one of three major isoforms of the SREBF transcription factor family, which activates genes involved in the synthesis of cholesterol, fatty acids, and triglycerides, whereas the SREBP-1c isoform predominantly regulates genes involved in the de novo lipogenesis (DNL) pathways [4,5,6]. The transgene includes the N-terminal active domain of the human SREBP-1c, under the control of the liver-specific albumin promoter (alb-SREBP-1c), which leads to constitutively active DNL in the liver [2]. It is relevant to mention that the fatty liver phenotype develops without the need of any dietary intervention. Further, in vivo studies revealed that the accumulation of lipids in the livers of these animals is associated with hepatic (but not systemic) insulin resistance [1]. Furthermore, the liver phenotype is persistent, and insulin resistance remains specific to liver tissue while the animals age [1]. In addition, the hepatic transcriptome from alb-SREBP-1c mice suggests mitochondrial dysfunction as a significant pathway affected in knowledge-based transcriptome analyses [3].

In humans, metabolic dysfunction-associated fatty liver disease (MAFLD) is a metabolic condition where a fatty liver phenotype is present in combination with chronic conditions, like hyperglycemia, hyperlipidemia, or hyperinsulinemia [7]. MAFLD can range from the simple accumulation of lipids in liver tissue, termed simple steatosis, to more severe forms of liver disease such as steatohepatitis or cirrhosis [8]. The contribution of mitochondrial function to MAFLD pathology remains controversial, with observations of either increased or decreased mitochondrial respiratory activity in diseased liver. Increased mitochondrial activity was predominantly observed in mice and men with simple steatosis, the early stage of MAFLD [9,10,11,12]. Interestingly, the increase in mitochondrial respiration significantly dropped when liver disease progressed to steatohepatitis [11]. This suggests that mitochondrial metabolism initially adapts to a shift in substrate availability in the liver to prevent lipotoxicity and liver damage. To date, the underlying molecular mechanisms leading to increased mitochondrial function in early stages of MAFLD remain largely unknown.

In our study, we investigated the respective mitochondrial electron flow and respiratory dynamics to elucidate the mechanisms behind increased mitochondrial function in simple steatosis. We used omics approaches combined with functional characterization of metabolic status in the livers of the alb-SREBP-1c mouse model. To identify possible changes in electron transport chain (ETC) components abundance and to identify the variations of protein abundances in central metabolic pathways for lipid, glucose, and glutamine metabolism, enriched mitochondria and lysates of hepatocytes were analyzed by proteomic approaches. In parallel, extracellular flux analyses were performed to investigate functional consequences for mitochondrial respiratory activity by the changes in subunit abundance of liver mitochondria. Moreover, enzyme assays were performed to assess hepatic substrate flows. We identified significant changes in abundance of ETC complex I and complex II subunits, which were accompanied by functional changes. We showed that mitochondrial respiratory function was significantly increased in the liver from the steatosis model, which was associated with a metabolic shift toward the oxidation of glucose, glutamine, and its metabolites.

## 2. Results

### 2.1. MAFLD and Mitochondrial Capacity

#### 2.1.1. Alterations of Protein Abundance in Mitochondrial ETC Complexes

**Figure 1:**
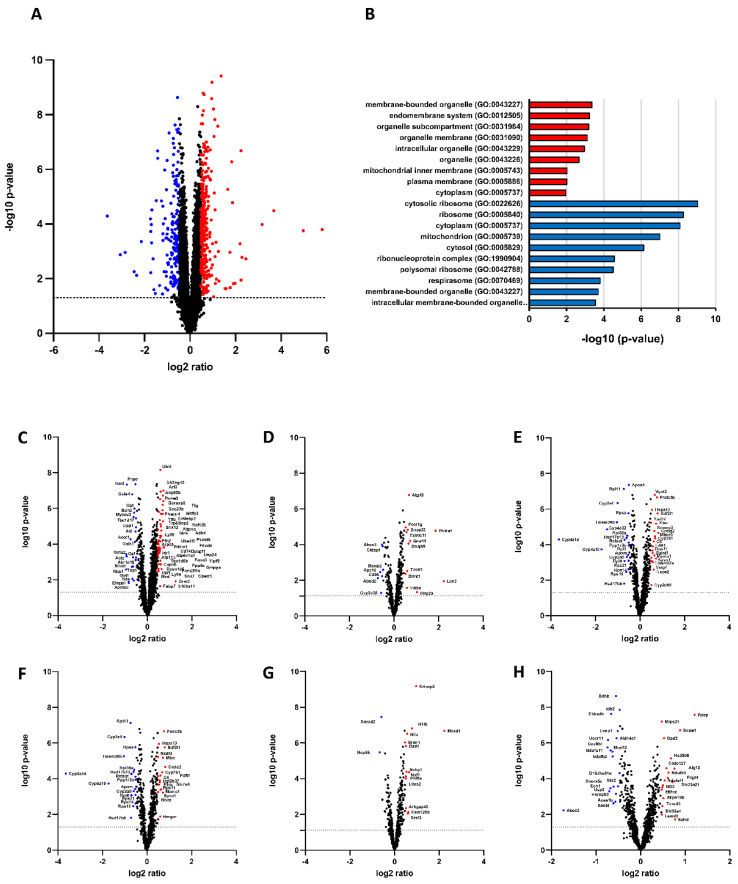
Impact of constitutive overexpression of SREBP-1c on hepatic transcriptome. (**A**) The log2 fold changes of coding and noncoding RNA abundance in alb-SREBP-1c vs. C57Bl6 mice (n = 8 animals per condition). 22,026 differentially abundant RNA species were detected. Upregulated (red; n = 314) or downregulated proteins (blue; n = 191) were determined by Student’s *t*-test (*p* > 0.05) with a 1.5-fold regulation. (**B**) RNA species regulated by alb-SREBP-1c were subjected to functional enrichment analyses. Significantly enriched pathways (FDR < 0.1) are shown for GO biological process ontology. A modified Fisher’s exact test was used for the functional enrichment analyses. *p* values were corrected for multiple testing using the Benjamini–Hochberg (FDR) method. (**C**–**H**) Transcriptomic signature of cellular compartments: (**C**) Cytoplasma, (**D**) Plasma membrane, (**E**) Endoplasmic reticulum and Golgi apparatus, (**F**) Endosome and Lysosome, (**G**) Nucleus, (**H**) Mitochondria and Peroxisomes.

The mitochondrial function in MAFLD has not yet been determined. RNA analyses of alb-SREBP-1c livers (Figure 1) revealed a strong change of the mitochondrial transcriptome (Figure 1H).

**Figure 2:**
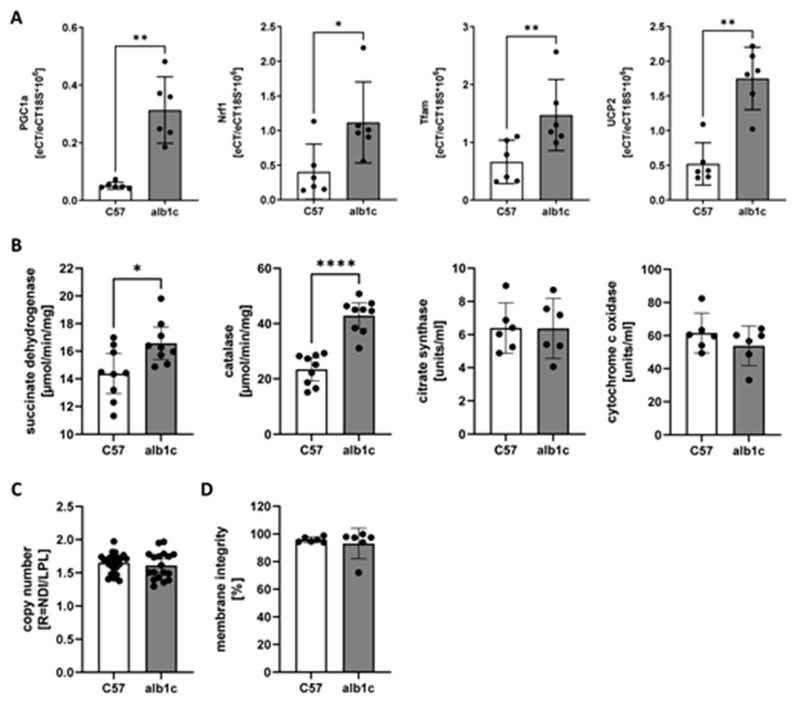
Mitochondrial gene expression and enzyme activities. (**A**) Expression of mitochondrial transcripts Pgc1a, Nrf1, Tfam and Ucp2. (**B**) Enzyme activities of succinate dehydrogenase, catalase, citrate synthase and cytochrome c oxidase. (**C**) Mitochondrial DNA copy number in relation to genomic DNA. (**D**) Mitochondrial membrane integrity from fractions of enriched liver mitochondria used for functional analyses based on cytochrome c oxidase enzyme activities. Data are expressed as means (±95% CI, n = 6–24 per group). Statistics: Mann–Whitney test, * *p* < 0.05, ** *p* < 0.01, **** *p* < 0.0001.

The expression of mitochondrial transcripts PGC-1a, Nrf1, Tfam, and Ucp2 consistently increased in alb-SREBP-1c mice, whereas the mitochondrial (mt)DNA copy number showed no differences between the two animal models (Figure 2A,C). Succinate dehydrogenase (Sdh) and catalase activity were increased, but not cytochrome c oxidase and citrate synthase (Figure 2B,D).

**Figure 3:**
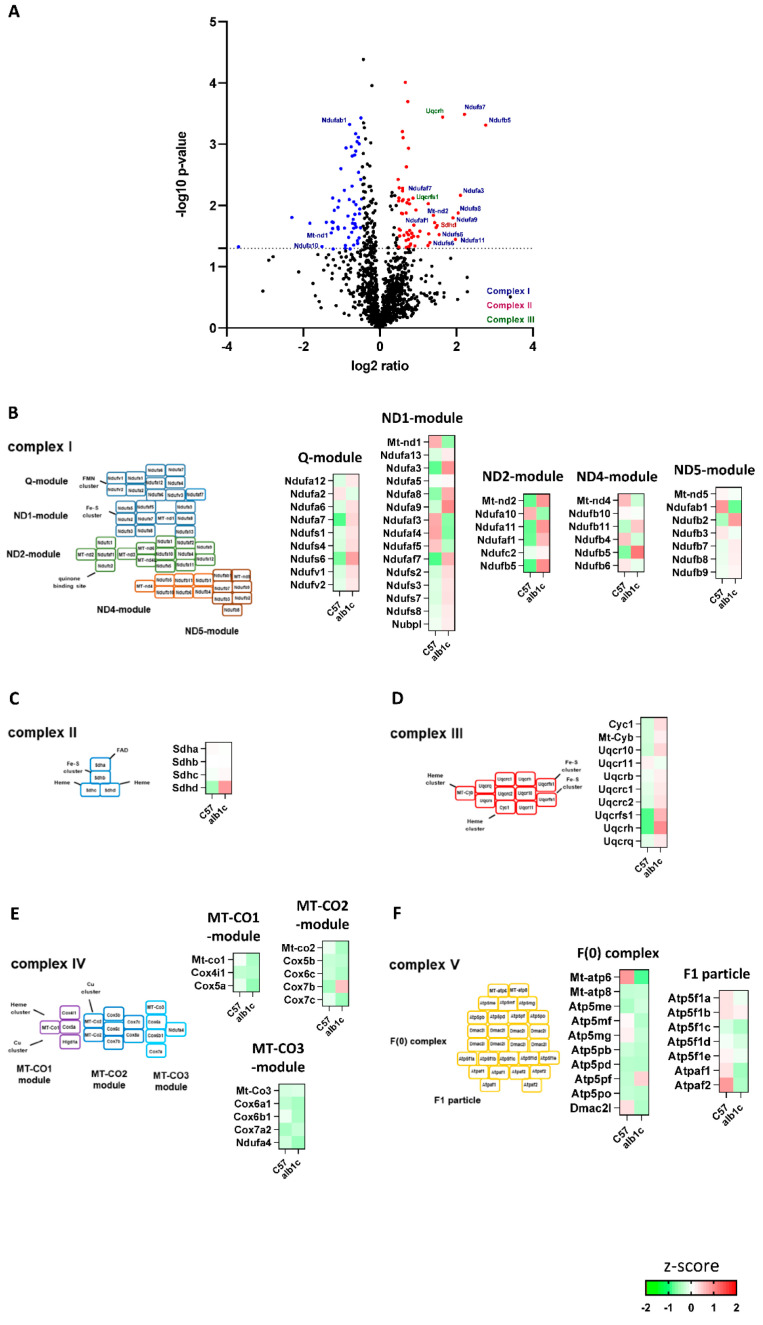
Regulated mitochondrial proteins in early stage of MAFLD. (**A**) The log2 fold changes of protein abundance in fractions of enriched mitochondria from alb-SREBP-1c vs. C57Bl6 liver tissue (n = 6 animals per condition). 1424 proteins were detected. Upregulated (red; n = 48) or downregulated proteins (blue; n = 40) were determined by Student’s *t*-test (*p* > 0.05) with at least 1.5-fold regulation. (**B**–**F**) Z-score analyses of protein abundance for individual complex I, II, III, IV, and V subunits: Red identifies upregulation of proteins, and green identifies downregulation. White identifies no differences.

To perform an unbiased analysis of mitochondrial protein composition as a potential predictor of MAFLD, we analyzed the mitochondrial proteome of isolated mitochondria from alb-SREBP-1c compared with C57Bl6 livers. In a label-free proteome analysis approach, a total of 1424 proteins were identified. Of these, 88 proteins showed significantly altered abundance in alb-SREBP-1c mitochondria (upregulated proteins: 48/downregulated proteins: 40; ratio: >1.5; *p*-value: <0.05). Of the 96 described mouse ETC proteins [13], 89 were identified here, including 17 differentially regulated. Broken down to the individual complexes, proteins of complexes I, II, and III showed significant differences in abundance (CI: n = 14 regulated/n = 43 identified; CII:1/4; CIII: 2/10) (Figure 3A). In the respiratory complex I, the main changes in protein abundance were found in three of five modules, i.e., Q- (Ndufa3, Ndufa8, Ndufa9, Ndufaf7), ND1- (Ndufs6 Ndufa7, Mt-nd1), and ND2-module (Ndufaf1, Ndufa11, Ndufs5, Mt-nd2 Ndufa10) (Figure 3B). The succinate dehydrogenase (complex II) showed significantly increased abundance solely for subunit d (Sdhd) in the alb-SREBP-1c model (Figure 3C). Ubiquinol cytochrome c oxidoreductase (complex III) showed a statistically significant increased abundance of two subunits, namely, the Rieske Fe–S protein (Uqcrfs1) and Uqcrh (Figure 3D) in mitochondria derived from alb-SREBP-1c, while no significant changes were found in the subunits of cytochrome c oxidase (complex IV) or ATP synthase (complex V) (Figure 3E,F).

#### 2.1.2. Altered ETC Subunit Abundance Interfered with Function

**Figure 4:**
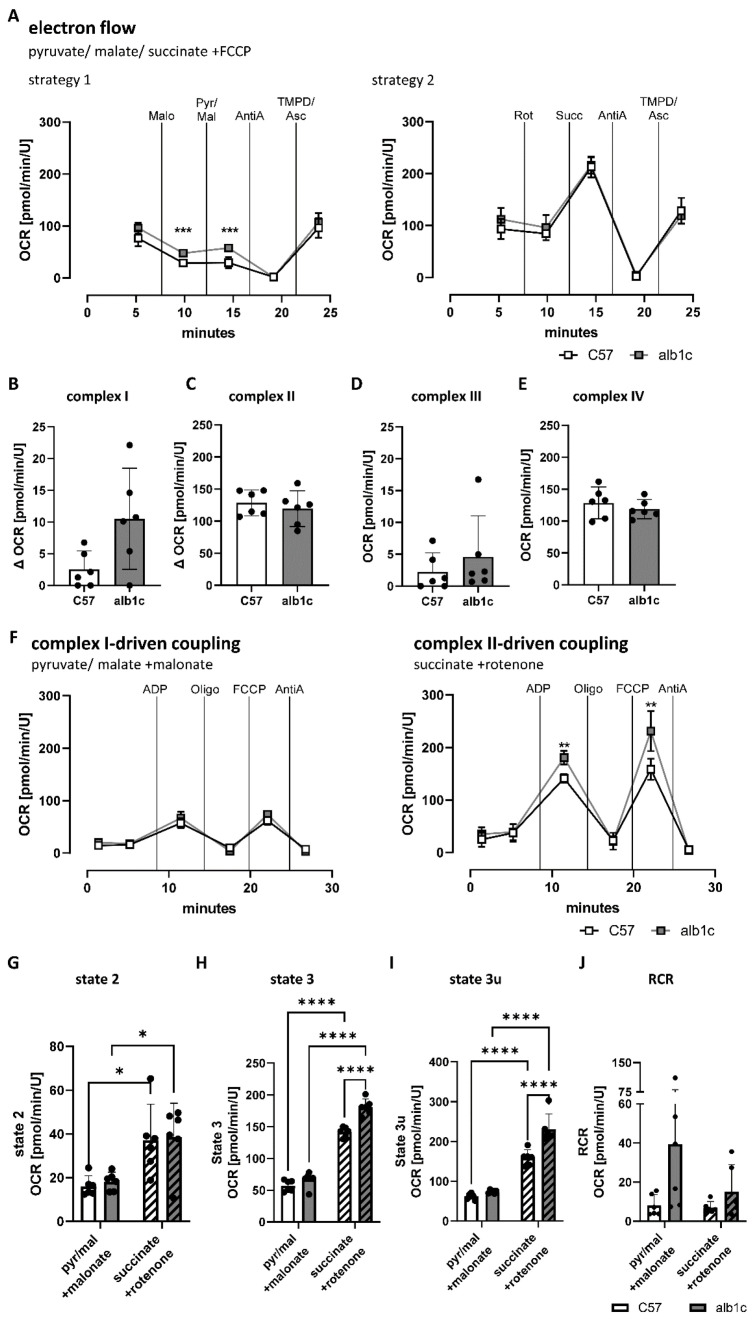
Electron flow capacity and coupling efficiency of mitochondrial ETC in alb-SREBP-1c mitochondria. Oxidative capacity was measured in response to manipulation of the electron transport chain (ETC) in fractions of enriched mitochondria from alb-SREBP-1c vs. C57Bl6 control livers. (**A**) The electron flow was assayed in the uncoupled state of the mitochondrial membrane with a combination of pyruvate, malate, and succinate as substrates for oxidation using two different injection strategies. Recorded oxygen consumption rates (OCR) over the time course of the assays are shown. (**B**) Complex I-specific OCR as difference between stimulation of complex I with pyruvate and malate and inhibition of complex II with malonate. (**C**) Complex II-specific OCR as difference between stimulation of complex II with succinate and inhibition of complex I with rotenone. (**D**) Complex III-specific OCR after inhibition with antimycin A and (**E**) complex IV-specific OCR after stimulation with TMPD/ascorbate. (**F**) Coupling experiments were conducted by measuring OCR individually for complex I- and II-driven mitochondrial coupling in fractions of isolated liver mitochondria. (**G**) Basal respiration (state 2), (**H**) oxidative phosphorylation (state 3), (**I**) maximal respiratory capacity (state 3u), and (**J**) respiratory control ratio were calculated. Data are expressed as means (±95% CI, n = 6 per group). Statistics: Mann–Whitney test or two-way ANOVA with Tukey’s multiple comparison, * *p* < 0.05, ** *p* < 0.01, *** *p* < 0.001, **** *p* < 0.0001. Malo: Malonate, Pyr/Mal: Pyruvate and Malate, AntiA: Antimycin A, TMPD/Asc: TMPD and Ascorbate, Rot: Rotenone, Succ: Succinate, Oligo: Oligomycin, C57: C57Bl6, alb1c: alb-SREBP-1c.

The impact of changes in mitochondrial subunit abundance were further analyzed for potential changes in the capacity of complex I to IV to transfer electrons within the ETC. Electron flows were assessed by measurement of the oxygen consumption rate (OCR) in mitochondrial fractions from alb-SREBP-1c compared with C57Bl6 livers in the presence of a combination of complex I- and II-specific substrates, namely, pyruvate, malate, and succinate, that were present in the assay medium with uncoupled mitochondria by adding FCCP (Figure 4). Two different strategies were applied to analyze electron flow: Strategy 1 included initial inhibition of complex II by malonate, followed by stimulation of complex I with pyruvate and malate, while strategy 2 started with inhibition of complex I by rotenone prior to stimulation of complex II with succinate. In both strategies, following the stimulation of complex I or II, complex III was inhibited with antimycin A, and complex IV was stimulated with TMPD/ascorbate during further course of the assay (Figure 4A). Complex I-specific OCR was then defined as the measurement after stimulation of complex I under complex II inhibition (Figure 4A,B), and complex II-specific OCR was defined as the measurement after stimulation of complex II under complex I inhibition (Figure 4A,C). In uncoupled state, the complex I-specific electron flow showed increased mean OCR in alb-SREBP-1c mitochondria, which failed to reach statistical significance (Figure 4B). No differences were found for complex II, III, and IV electron flow activity between alb-SREBP-1c and control mitochondria (Figure 4C–E).

Next, coupling efficiency was investigated individually for specific complex I-driven or complex II-driven mitochondrial OCR (Figure 4F–J). In this assay, specific complex I- or II-driven basal oxygen consumption (state 2), maximal oxidative phosphorylation in response to the addition of ADP (state 3), and maximal respiratory capacity induced by uncoupling of mitochondrial membrane with FCCP (state 3u) were assessed. Complex I-specific substrates, pyruvate in combination with malate, and simultaneous inhibition of complex II using malonate were applied to investigate complex I-specific activity (Figure 4F). No changes in complex I-driven respiration were observed over the time of the assay between alb-SREBP-1c and control group (Figure 4F). In contrast, specific complex II-driven mitochondrial activity, using succinate as complex-specific substrate in combination with rotenone as complex I inhibitor, showed significantly increased respiratory activity after ADP injection to induce state 3 and after FCCP injection to induce state 3u in alb-SREBP-1c mitochondria (Figure 4F,H,I). Direct comparison of complex I-driven and complex II-driven mitochondrial OCR showed an overall two- to threefold increase in complex II-driven respiration in liver mitochondria (Figure 4G–I). State 2 remained unchanged between alb-SREBP-1c and control mitochondria, while the complex II-driven state 3 and state 3u were significantly increased by 28% and 46% in the alb-SREBP-1c model, respectively (Figure 4G–I). Respiratory control ratio (RCR) was calculated as the ratio of ADP- to oligomycin-stimulated (complex V inhibition) OCR measurements (Figure 4J). Both complex I-driven and complex II-driven RCR tended to be increased in alb-SREBP-1c mitochondrial respiration, suggesting increased coupling efficiency in alb-SREBP-1c compared to control mitochondria (Figure 4J).

#### 2.1.3. Increased Mitochondrial Function Related to Physiological Changes in Cellular Metabolism

**Figure 5:**
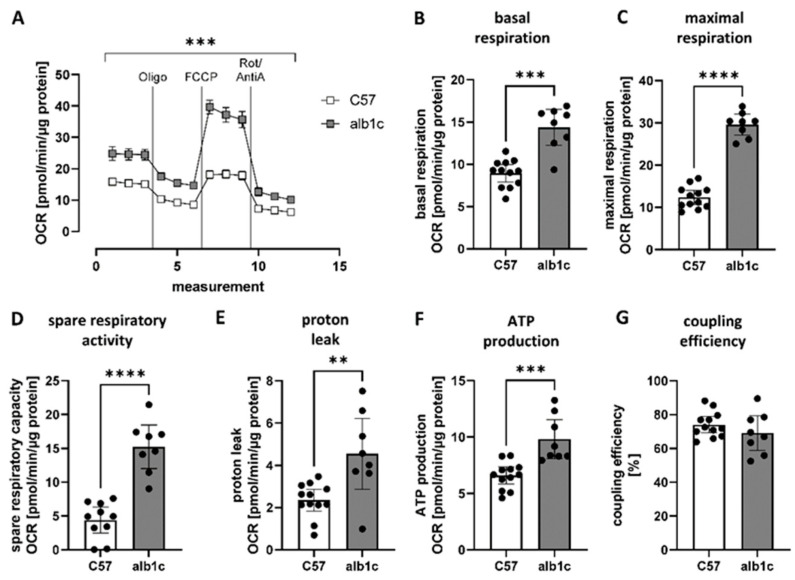
Characteristics of mitochondrial activity in physiological context. Mitochondrial function was measured in the physiological context of the cell in primary hepatocytes isolated from alb-SREBP-1c vs. C57Bl6 livers. (**A**) Mitochondrial respiration was measured in response to stimulation of electron transport chain complexes and calculation of (**B**) basal and (**C**) maximal respiration, (**D**) spare respiratory capacity, (**E**) proton leak, (**F**) ATP production, and (**G**) coupling efficiency. Data are expressed as mean (±95% CI, n = 8–12 per group). Statistics: Mann–Whitney test, ** *p* < 0.01, *** *p* < 0.001, **** *p* < 0.0001. Oligo: Oligomycin, Rot/AntiA: Rotenone and Antimycin A, C57: C57Bl6, alb1c: alb-SREBP-1c.

Further, to assess the molecular link between SREBP-1c-forced metabolic changes present in hepatic simple steatosis and the alterations found in mitochondrial subunit abundance and function, we investigated the standard parameters of mitochondrial function in the physiological context of the cell. Cultured primary hepatocytes isolated from the animal models were analyzed in a serum-free standard culture medium without any inhibitions. The course of the assay is depicted in Figure 5A and showed an overall significantly increased OCR in hepatocytes from alb-SREBP-1c livers compared with controls. Calculated parameters of mitochondrial activity reflected significantly increased basal (1.6-fold, *p* < 0.001) (Figure 5B) and maximal respiration rates (2.4-fold, *p* < 0.001) (Figure 5C), as well as increased spare respiratory capacity (3.5-fold, *p* < 0.001) (Figure 5D), proton leak (1.9-fold, *p* < 0.01) (Figure 5E), and ATP production (1.5-fold, *p* < 0.001) (Figure 5F), while coupling efficiency remained unchanged (Figure 5G). The results indicated that not only was mitochondrial activity activated per se, it was even further increased in the physiological context, suggesting that metabolites of changed hepatic metabolism additionally affect mitochondrial activity in SREBP-1c-forced simple hepatic steatosis.

### 2.2. Mechanisms of Mitochondrial and Energy Metabolism in Early-Stage MAFLD

#### 2.2.1. Impact of SREBP-1c-Forced DNL in Early-Stage MAFLD on Transcriptome of Primary Hepatocytes

To address the molecular mechanisms of changed hepatic metabolism affecting the observed changes in mitochondrial and energy metabolism of SREBP-1c-forced simple hepatic steatosis, we focused on isolated hepatocytes. Gene-expression analyses identified 125 downregulated and 279 upregulated annotated transcripts in alb-SREBP-1c hepatocytes (Supplementary Table S1, dataset A). As in the liver, targets of the Srebf-1 gene were enriched (5.98 × 10^−7^) with activation of downstream targets (1.764). Further upstream regulatory molecules deduced from the dataset were Ago (*p*-value of overlap: 1.97 × 10^−18^), indicating increased regulatory RNA expression and transcription factors (Ppara, 4.63 × 10^−10^; Pgc1a, 6.201 × 10^−9^; Cepba, 2.72 × 10^−7^) or Insr (2.01 × 10^−7^) and D-glucose (1.79 × 10^−7^) (Supplementary Table S2, dataset A). Overall, as in the liver, the differential expression strongly suggested mitochondrial dysfunction (log *p*-values of overlap: 4.23), but also ketogenesis (2.5) and ketolysis (4.28), indicating alterations in the metabolic substrate flow in hepatocytes (Supplementary Table S3 dataset A).

Moreover, in the expression analyses in hepatocytes, molecules with predicted activation scores > 2 were mainly miRNAs. Overall, 77 annotated miRNAs were differentially abundant in hepatocytes of alb-SREBP-1c and C57Bl6 mice (22 down- and 55 up-regulated in alb-SREBP-1c), including miR210 (Supplementary Table S1 dataset B; Supplementary Figure S1). On a cellular functional level, most molecules were involved in hepatic growth (hyperplasia; *p*-value overlap: 2.37 × 10^−4^) and related processes. A positive activation score could be deduced from the miRNA pattern for signaling pathways (Parn (1.982); Tgfß1 (1.964); Agt (1.414); Smad2/3 (1.28); Lats2 (1.414); Dnmt (1.414)) but also for insulin signaling (Igf1r (1.011); Insr (0.972)). A negative activation score was observed, e.g., for signaling-related genes (Bdnf (−1.98), Tnfrs1b (−0.966), or insulin (−1.067)). Differences in miRNA patterns may point to beginning hepatic hyperplasia and the fine-tuning of insulin signaling and glucose metabolism (Supplementary Table S2, dataset B).

#### 2.2.2. High Mitochondrial Activity in Early-Stage MAFLD Was Mainly Driven by SREBP-1c-Forced DNL and Glucose Oxidation

**Figure 6:**
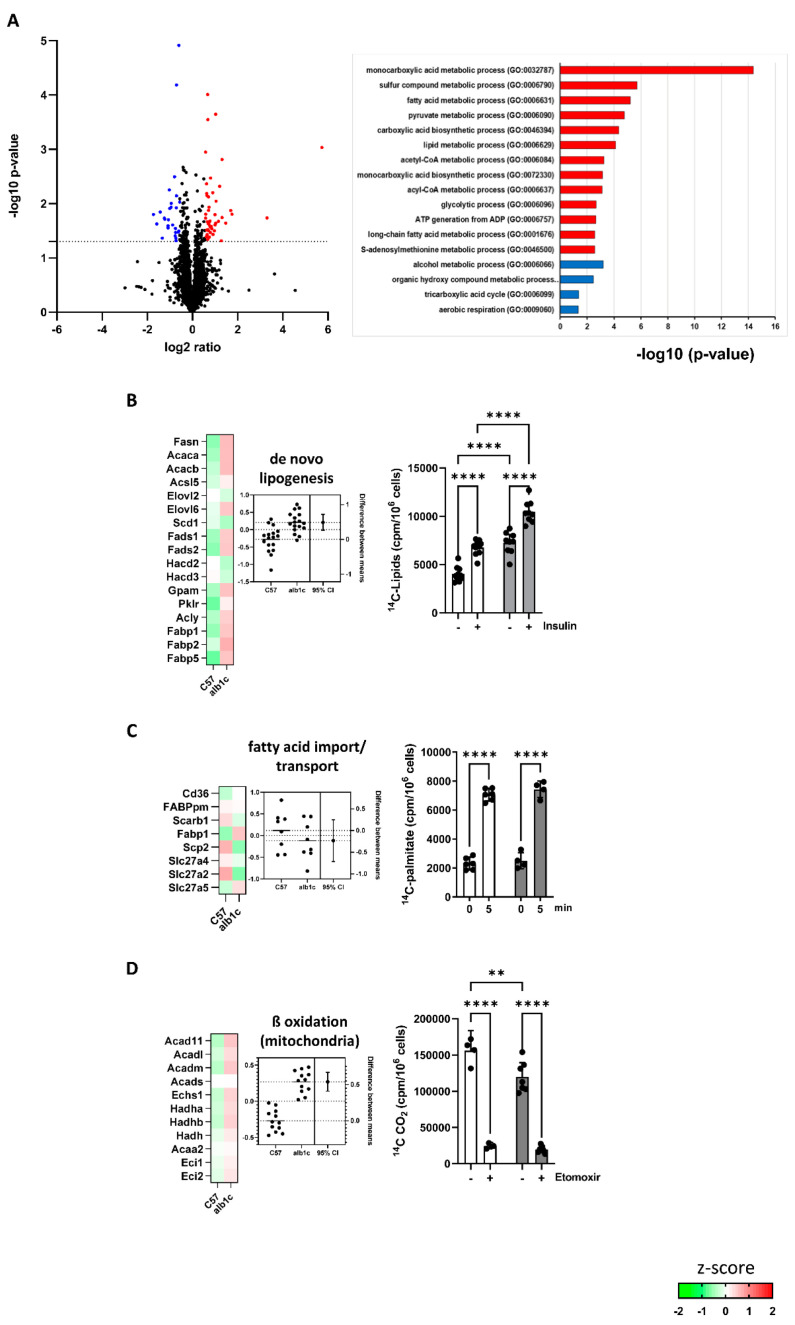
Changes in biosynthesis processes in early-stage MAFLD. (**A**) The log2 fold changes of protein abundance in alb-SREBP-1c vs. C57Bl6 hepatocytes (n = 6 animals per condition). 2731 differentially abundant proteins were detected. Upregulated (red; n = 46) or downregulated proteins (blue; n = 26) were determined by Student’s *t*-test, with criteria set to *p*-value < 0.05 and >1.5-fold regulation. Proteins regulated by alb-SREBP-1c were subjected to functional enrichment analyses. Significantly enriched pathways (FDR < 0.1) are shown for biological processes (GO). A modified Fisher’s exact test was used for the functional enrichment analyses. *p*-values are corrected for multiple testing using the Benjamini–Hochberg (FDR) method. (**B**–**D**) Heat maps resulting from z-score analyses and estimation plots for each pathway are included: (**B**) de novo lipogenesis, (**C**) fatty acid import/transport, (**D**) fatty acid β-oxidation (mitochondrial). Estimation plots show the differences between means of alb-SREBP-1c vs. C57Bl6 (±95% CI) (mean z-score: left axis, effect size: right axis). Functional analysis show data expressed as means (±95% CI, n = 3–6 per group—depending on the assay). Color code for assays (**B**–**D**): white bars, data from C57Bl6 mice and gray bars, data from alb-SREBP-1c mice. Statistics: Mann–Whitney test or two-way ANOVA with Tukey’s multiple comparisons, ** *p* < 0.01, **** *p* < 0.0001. C57: C57Bl6, alb1c: alb-SREBP-1c.

To follow the consequences of altered gene expression, the proteome of primary hepatocytes was analyzed. In a label-free proteome analysis approach with 6 animals per group, 2731 proteins could be identified. Of these, 72 proteins were significantly altered in their abundance in alb-SREBP-1c animals (upregulation: 46/downregulation: 26; ratio: >1.5; *p*-value: <0.05) (Figure 6A). Gene ontology analyses on biological processes indicated alterations in relevant substrate pathways of lipid and glucose metabolism (Figure 6A).

#### 2.2.3. Altered Protein Abundance of Main Lipid and Glucose Metabolism Pathways in Early-Stage MAFLD

Focusing the proteome data on glucose and lipid metabolism pathways, out of 315 related proteins, 255 different enzymes were identified and 21 of these were differentially abundant (upregulation: 13/downregulation: 8; ratio: >1.5; *p*-value: <0.05). The physiological consequences of these changes in protein abundance of key pathways from lipid and carbohydrate metabolism were investigated by enzyme activity assays. In alb-SREBP-1c mice, the pathways for de novo lipid synthesis (DNL) showed significantly upregulated activity maps deduced from differential abundant protein components (Figure 6B). Consistently, basal lipid synthesis indicated an 80% increase of DNL in alb-SREBP-1c animals compared to the controls, in vitro. In the alb-SREBP-1c-model, DNL still responded to insulin with 60% increase compared with insulin-stimulated cells from the controls (Figure 6B). Altered protein abundances in fatty acid import and transport resulted overall in unaffected activity map. This was consistent with the unchanged cellular palmitate uptake in alb-SREBP-1c and control group (Figure 6C). Protein analyses estimated that mitochondrial ß-oxidation was upregulated in alb-SREBP-1c mice. This could not yet compensate the in vitro oxidation of palmitate that was 30% decreased in alb-SREBP-1c hepatocytes (Figure 6D). Nevertheless, blocking the import of long-chain fatty acids into mitochondria with etomoxir showed that the difference in palmitate oxidation observed could be solely attributed to mitochondrial oxidative activity (Figure 6D).

**Figure 7:**
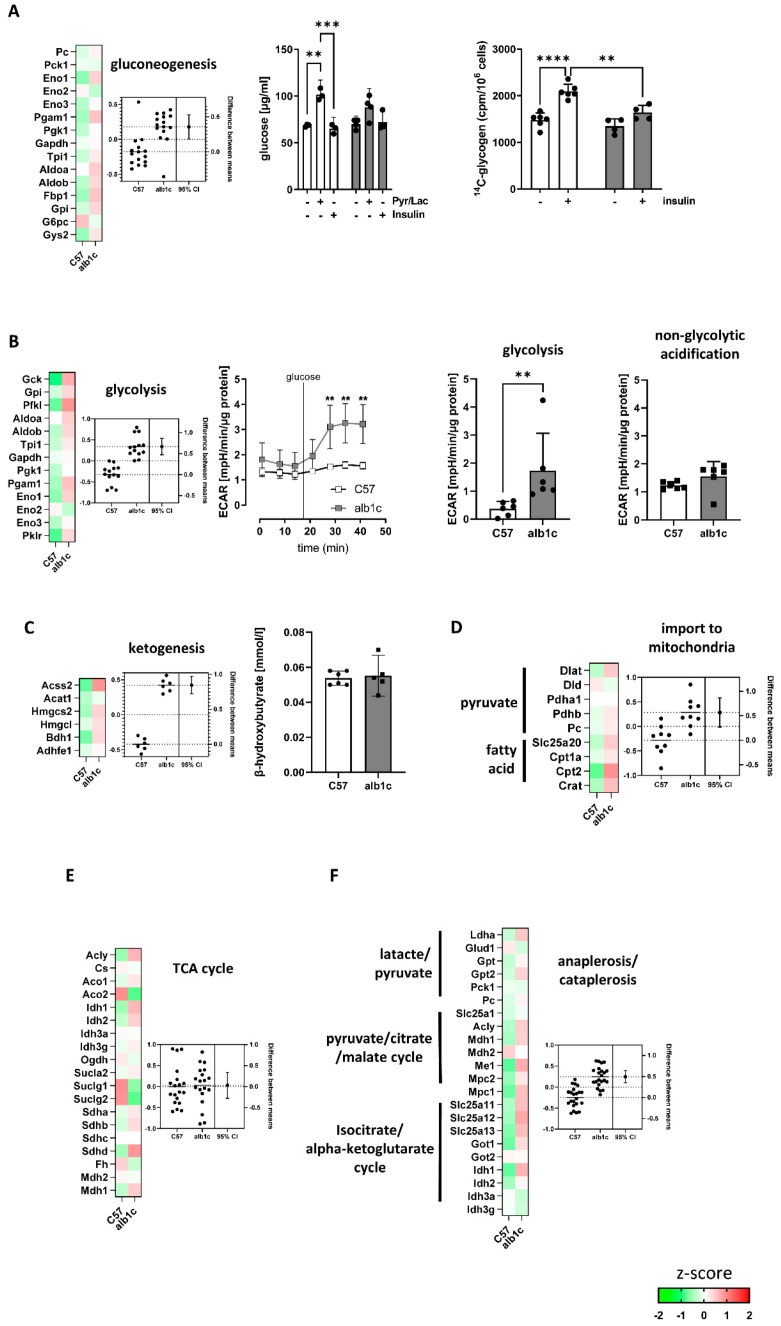
Changes in biosynthesis processes in early-stage MAFLD. (**A**–**F**) Heat maps resulting from z-score analyses and estimation plots for each pathway are included: (**A**) gluconeogenesis and glycogen synthesis, (**B**) glycolysis, (**C**) ketogenesis, (**D**) import to mitochondria, (**E**) TCA cycle, and (**F**) anaplerosis/cataplerosis. (**A**–**F**) Functional analysis of metabolic activities was investigated in primary hepatocyte cultures isolated from alb-SREBP-1c vs. C57Bl6 livers. (**C**) β-OH butyrate was analyzed in liver tissue. Estimation plots show the differences between means of alb-SREBP-1c vs. C57Bl6 (±95% CI) (mean z-score: left axis, effect size: right axis). Functional analysis show data expressed as means (±95% CI, n = 3–6 per group, depending on the assay). Statistics: Mann–Whitney test or two-way ANOVA with Tukey’s multiple comparisons, ** *p* < 0.01, *** *p* < 0.001, **** *p* < 0.0001. C57: C57Bl6, alb1c: alb-SREBP-1c, Pyr/Lac: pyruvate, lactate.

The analyses of gluconeogenesis and glycolysis showed an overall upregulation of proteins related to these pathways in alb-SREBP-1c animals (Figure 7A,B). However, focusing on the rate-limiting enzymes for gluconeogenesis, namely, pyruvate carboxylase (Pc), phosphoenolpyruvate carboxylase (Pck1), fructose-1,6-bisphosphatase (Fbp1), and glucose-6-phosphatase (G6pc). No significant increase was found in alb-SREBP-1c mice. Gluconeogenesis and glycogen synthesis following substrate or insulin induction were essentially unaltered, but insulin action was muted, as expected (Figure 7A). In contrast, glycolysis was considered as upregulated as the key enzymes, glucokinase (Gck), 6-phosphofructokinase (Pfkl), and pyruvate kinase (Pklr) showed significantly increased protein abundance in the alb-SREBP-1c (Figure 7B). The estimated increase in glycolytic activity was confirmed with a four-fold increase in glucose-stimulated glycolysis rate in the alb-SREBP-1c animal model compared to C57Bl6 controls (Figure 7B). The ketogenesis pathway was upregulated in alb-SREBP-1c on transcription and proteome levels. However, the abundance of the end product of β-hydroxybutyrate as a measure for ketogenesis did not differ between the groups (Figure 7C). Pyruvate and the fatty acid import to mitochondria as an initial part of the energy metabolism pathways as well as the urea cycle were upregulated in alb-SREBP-1c animals (Figure 7D and Supplementary Figure S2). Further, the TCA cycle components showed no differences in activity maps between both mouse models (Figure 7E). The basal non-glycolytic acidification rate tended to be increased in alb-SREBP-1c hepatocytes (Figure 7B), and the citrate synthase activity representing the rate-limiting enzyme in TCA cycle showed no difference between the two groups, supporting the TCA cycle proteome results (Figure 1B and Figure 7E). In contrast, the ana- and cataplerosis-associated proteins, especially isocitrate-alpha-ketoglutarate-cycle, showed marked upregulation in alb-SREBP-1c mice (Figure 7F).

Next to alterations in key metabolic lipid and glucose metabolic pathways, the proteome analyses indicated increased activity maps in intracellular substrate flux pathways in SREBP-1c-forced fatty liver. Proteins associated with glutamine metabolism, especially glutaminolysis, and urea cycle were upregulated in alb-SREBP-1c mice (Supplementary Figure S2). In contrast to that, the pathway maps for sterol biosynthesis, bile acid synthesis, triglyceride synthesis, VLDL assembly, sphingolipid metabolism, CDP-diacylglycerol synthesis, omega oxidation, alpha oxidation, peroxisomal ß-oxidation pentose phosphate, and alanine/pyruvate cycle showed no differences between both mouse models (Supplementary Figure S2).

#### 2.2.4. A Shift in Substrate Flux Pathways and Preferences Accompanied Early Stage MAFLD

**Figure 8:**
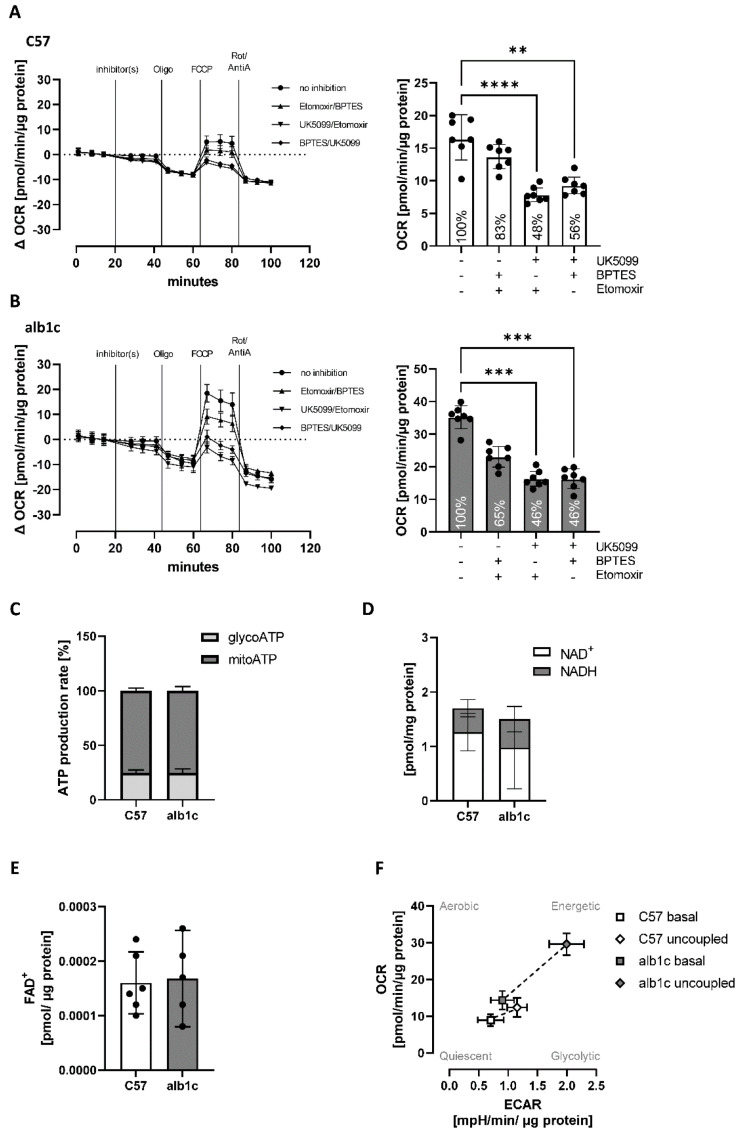
Substrate oxidation preferences and energetic profile of mitochondrial activity in SREBP-1c-forced early-stage MAFLD. (**A**,**B**) Substrate oxidation was measured by extracellular flux analyses in the absence or presence of the inhibitors UK5099, BPTES, and etomoxir to force substrate utilization specifically through glucose (etomoxir/BPTES), glutamine (UK5099/etomoxir) or long-chain fatty acids (UK5099/BPTES). Assay profiles for (**A**) C57Bl6 vs. (**B**) alb-SREBP-1c hepatocytes and calculated maximal oxygen consumption rates (OCR) for each condition tested are shown (bar charts). (**C**) ATP production rates were calculated as a percentage of the whole ATP production in primary hepatocytes from both phenotypes. (**D**) NAD^+^ and NADH as well as (**E**) FAD^+^ abundance were measured in mitochondrial fractions isolated from liver tissue of both phenotypes. (**F**) Energy phenotypes were assessed by measuring OCR vs. extracellular acidification rate (ECAR) in the primary hepatocyte culture from alb-SREBP-1c vs. C57Bl6 livers in basal and uncoupled states. Data are expressed as mean (±95% CI or ±SD (F), n = 5–12 per group—depending on assay). Statistics: ANOVA with Kruskal–Wallis test, ** *p* < 0.01, *** *p* < 0.001 **** *p* < 0.0001.

Substrate oxidation preferences were investigated in vitro using extracellular flux analyses (Figure 8). To measure single substrate-specific oxidation, hepatocyte OCR was measured without and with the exposure of primary hepatocytes to inhibitors that selectively block the utilization of glucose (UK5099), glutamine (BPTES), or long-chain fatty acids (etomoxir) for mitochondrial maximal oxidation. Glucose oxidation alone achieved 83% of maximal respiration in C57Bl6 but only 65% in alb-SREBP-1c hepatocytes (Figure 8A,B). Glutamine oxidation alone remained unchanged with 48% and 46% of maximal respiration, respectively (Figure 8A,B). Oxidation of long-chain fatty acids reached 56% of maximal respiration in C57Bl6 but 46% in alb-SREBP-1c hepatocytes (Figure 8A,B). The ATP production was derived approximately 25% from glycolysis and 75% from mitochondrial oxidative phosphorylation, in both groups (Figure 8C). Additionally, the level of energetic molecules NAD^+^, NADH, and FAD^+^ in mitochondrial fractions remained unchanged (Figure 8D,E). Nevertheless, the alb-SREBP-1c model showed increased energetic potential with a more glycolytic phenotype compared to the C57Bl6 control group, which was already present at basal level and became more pronounced when the cells were stressed by uncoupling of the mitochondrial membrane for maximal mitochondrial capacity (Figure 8F). Here, the energy phenotype of alb-SREBP-1c hepatocytes was estimated by plotting the oxygen consumption (OCR), as a measure of mitochondrial activity, and extracellular acidification (ECAR), as measure for glycolytic activity, for untreated (basal) and uncoupled respiration in comparison to the C57Bl6 control group (Figure 8F).

## 3. Discussion

Mitochondrial dysfunction is a common condition described in metabolic dysfunction-associated fatty liver disease (MAFLD). In this study, we were able to shed light on the molecular mechanisms behind increased mitochondrial function in a mouse model resembling the key features of MAFLD, i.e., a moderate increase in hepatic lipid content with selective hepatic insulin resistance [1,2]. In this model, the DNL-driven mild hepatic steatosis was accompanied by (i) significant changes in the mitochondrial transcriptome and proteome, (ii) altered abundance of respiratory chain complexes I and complex II subunits with impact on electron flux, (iii) altered hepatocyte proteome with impact on key lipid and glucose metabolism pathways indicating differences in substrate flux and (iv) a shift in substrate utilization towards a glycolytic phenotype.

The development of hepatic steatosis is mainly driven by SREBP-1c [6,14], supported by the observation that up to 38% of hepatic triglycerides are derived from DNL in this pathology [15]. In patients with metabolic dysfunction, such as MAFLD, a phenomenon called selective hepatic insulin resistance occurs. When this happens, SREBP-1c is still responsive to insulin to activate DNL, but insulin fails to inhibit hepatic gluconeogenic pathways [16,17]. Therefore, the transgenic alb-SREBP-1c mouse model, with constitutively active DNL, due to liver-specific overexpression of hSREBP-1c and hepatic insulin resistance [1,2], represented an adequate model system to analyze the underlying molecular mechanisms of mitochondrial function observed in the early stage of human MAFLD.

### 3.1. Mechanisms in Early-Stage MAFLD to Alter Mitochondria and Energy Metabolism

The anterograde and retrograde regulation of mitochondrial biogenesis, mitophagy, mtDNA transcription, and replication is highly dynamic and tightly controlled to ensure adaption to cellular demands. Tfam, Nrf1, or PGC-1α and its gene regulatory network were increased in alb-SREBP-1c mice. Tfam acts as histone on mtDNA, regulating transcription and replication [18,19]. The transcriptional co-activator PGC-1α plays a multifaceted role to regulate mitochondrial metabolic functions and mitochondrial turnover [20,21,22]. PGC-1α is a direct transcriptional target of SREBP-1c [23] and regulates the expression and interaction of the transcription factors Nrf1 and Nrf2 [22,23,24] while also regulating (mt)DNA transcription and replication [24]. Nrf1 further promotes the nuclear expression of key mitochondrial transcription and translation machinery components to regulate mitochondrial mass and oxidative phosphorylation capacities [24].

In the alb-SREBP-1c mouse model, no changes of mitochondrial copy numbers were observed ([2], our study), indicating other cellular mechanisms contributed to the increased mitochondrial activity found in early fatty liver pathology. The succinate dehydrogenase subunit d promoter region contained a Nrf1-promoter element within 150bp proximity to the transcription start site, suggesting a direct transcriptional regulation [25]. This may be supported by the increased protein abundance and enhanced succinate dehydrogenase activity observed in this study. In line with this, the SREBP-1c activation could be considered to exert direct transcriptional regulation on mitochondrial turnover to adapt to metabolic demands.

As another key metabolic transcriptional regulator, the genes of the PPARa regulatory network were activated in the livers of the alb-SREBP-1c mice [3] and hepatocytes in our study. Recent studies from our group showed that in various mouse models with fatty liver phenotype, alterations in proteome were not restricted to mitochondria. Altered peroxisomal protein patterns were also associated with SREBP-1c expression in fatty livers [26,27]. Peroxisomes are involved in the oxidation of complex fatty acid structures or cholesterol synthesis for release as bile acids to counteract the accumulation of excess fatty acids in liver tissue [26,27,28,29,30,31,32]. The coordination of these organelles was further described to be induced by the PPARa axis [33], which was identified to be activated in the livers of the alb-SREBP-1c mice [3]. Activated peroxisomal oxidation might serve to provide smaller acyl-CoA as substrate for mitochondrial oxidative phosphorylation and therefore contribute to increased mitochondrial activity in this stage of liver disease to maintain the cellular energy level. The increase of peroxisomal mass in fatty liver of alb-SREBP-1c mice might also be the cellular response to reduce the lipotoxic burden of the cell by increased activation of cholesterol synthesis to release fatty acids via bile acid [27,34]. In addition, the increased catalase activity supported the point of view that, next to anabolic pathways, peroxisomes act protectively to counteract accumulation of the reactive oxygen species due to the increased oxidative activity, especially in early stage of fatty liver [35].

In addition to mitochondria-related gene expression patterns, expression analysis revealed an overrepresentation of differentially abundant miRNAs in the fatty liver of SREBP-1c mice. This may indicate an immediate–early response to activate hepatic regeneration processes, activate cell communication, and counteract metabolic distress. The miR210 is involved in apoptosis, hypoxia, and gene regulation. It has been shown to promote hepatic glycolysis and mitochondrial respiratory capacity [36].

The role of the various metabolic pathways, including glycolytic pathways in NAFLD and MAFLD, was recently reviewed [37,38,39]. Transcriptional analyses and meta-analyses thereof have been performed in humans with various stages of NAFLD in comparison to healthy controls or obese patients. Gene transcriptional alterations associated with insulin receptor function/glucose metabolism, lipid metabolism, and PPARA signaling, including direct SREBP1-c and PGC-1A targets [40,41,42,43,44,45], have been shown. Furthermore, a recent study also identified mitochondrial genes such as MT-CO1, NDUFA11, NDUFA4L2, ETHE1, and GDPD1 to be differentially regulated on the expression level as in our study [46]. In addition, a common gene variant in PGC-1A affecting PGC1A levels were associated to the development of NAFLD [47]. This may further support our observations.

### 3.2. MAFLD Is Accompanied by a Shift in Substrate Flux Pathways and Preferences

Respiratory chain complexes I and II represent independent entry sites for electrons derived from cellular substrate breakdown to the respiratory chain. Hence, they also provide a direct link between mitochondrial activity and cellular metabolism. Our data revealed an altered protein abundance of complex II subunit d and succinate oxidation via complex II resulted in significantly increased respiration in mitochondria from the alb-SREBP-1c steatosis model. The increased activity in enriched mitochondria fractions was more pronounced within the physiological context of the hepatocyte. This suggests that changes in cellular metabolism considerably contribute to changes in mitochondrial respiration in DNL-driven steatosis, supported by significantly changed abundance of key enzymes from metabolic pathways in proteome analyses.

In this study, the remodeling of respiratory chain proteins with increased mitochondrial activity was accompanied by significant changes in hepatic metabolism. Hepatocyte transcriptome analyses indicated changes in substrate flows as main targets of differential gene expression. This was further supported by proteome analysis, indicating changes in pathways regulating carbohydrate and fatty acid metabolism. In detail, DNL and glycolysis showed increased activity, while fatty acid oxidation was decreased. An increase in glycolytic activity led to the accumulation of malonyl-CoA, which is a potent inhibitor of carnitine palmitoyltransferase 1 (CPT1), thus regulating carnitine-dependent fatty acid transport into mitochondria for oxidation [9,48,49]. Therefore, decreased fatty acid oxidation in fatty liver of alb-SREBP-1c mice might be, at least in part, induced by the marked increase in glycolytic activity. In turn, the increase in glycolytic activity most likely represents a cellular response to the increased SREBP-1c-forced DNL rate, to provide sufficient carbon sources for lipid synthesis.

Glycolysis fuels lipid synthesis by the conversion of glucose to pyruvate. Pyruvate enters the mitochondria to fuel the TCA cycle to produce citrate, which is redirected to the cytosol to be converted into acetyl-CoA as substrate for DNL instead to contribute to the TCA cycle [50]. As a result, high rates of SREBP-1c-forced DNL require high concentrations of carbon sources mainly provided by glucose; this enhances a shift of substrates for mitochondrial oxidation to alternate substrate sources. Further, this implication was supported by the results of fuel oxidation experiments, where glucose presented the predominant substrate oxidized in extracellular flux analyses in alb-SREBP-1c hepatocytes.

In addition, the omics analyses indicated increased glutaminolysis, ketogenesis, and changes in the isocitrate-α-ketoglutarate cycles in fatty liver of alb-SREBP-1c mice, further supporting a metabolic shift of substrate sources for oxidative activity. Although ketogenesis showed increased activity in liver proteome analysis, the β-hydroxybutyrate levels in liver were unaltered. Fatty acid oxidation presents the major source of ketogenesis. However, in human steatosis, ketogenesis was negatively correlated with hepatic triglyceride content, and fatty acid oxidation derived acetyl-CoA was primarily diverted into TCA cycle [51]. These data are in line with the decreased fatty oxidation found in the alb-SREBP-1c model, suggesting that the resulting amounts of acetyl-CoA from fatty acid oxidation mainly fuel citrate synthesis to provide substrate for SREBP-1c-forced DNL.

The metabolic changes observed in fatty liver of alb-SREBP-1c mice collectively point towards the adaption of substrate flows to SREBP-1c-forced DNL and simultaneously forcing a shift in substrate sources for mitochondrial oxidative phosphorylation. Consequently, the mitochondrial oxidative phosphorylation machinery adapts in response to the changes in liver metabolism of alb-SREBP-1c mice to fulfill the energy demand of the cell. Our data show that succinate oxidation via complex II is the predominant site of increased mitochondrial action. This adaption of the mitochondrial machinery seems to be the result of the increased acetyl-CoA demand for SREBP-1c-forced DNL, which in turn reduces the acetyl-CoA available to enter the TCA cycle as substrate for full respiratory chain activity. To maintain substrate flows toward the mitochondria, compensation via glutamine-derived anaplerosis through isocitrate-α-ketoglutarate cycles fuels the TCA cycle, shifting the mitochondrial electron flow predominantly through complex II [52].

## 4. Materials and Methods

### 4.1. Animals

C57Bl6 (C57Bl6) and B6-TgN(alb-HA-SREBP-1cNT) (alb-SREBP-1c) [2] mice with liver-specific overexpression of transcriptionally active human SREBP-1c (aa 1-436) were bred and maintained under standard conditions (12 h light/dark cycle; 22 °C ± 1 °C, 50% ± 5% humidity). At 6 weeks of age, male littermates of each genotype were kept under standardized conditions with free access to water and regular laboratory chow (13.7 mJ/kg: 53% carbohydrate, 36% protein, 11% fat (Ssniff, Germany)). Male mice between 18 to 24 weeks of age were dispatched by CO_2_ asphyxiation before the removal of liver tissue or isolation of primary hepatocytes for analyses. Liver tissue for RNA/DNA analyses was snap-frozen in liquid nitrogen and stored at −80 °C until further processing. The hepatic lipid accumulation in alb-SREBP-1c mice [1,2] was also verified for the animals used in this study by increased total fatty acids compared to C57Bl6 mice (Supplementary Table S3). Animal experiments were approved (LANUV, NRW, Germany, Az.84-02.04.2015.A424) and performed in accordance with the German law on animal protection and the ‘Principle of laboratory animal care’ (NIH publication No. 85-23, revised 1996).

### 4.2. Hepatocyte Analyses

#### 4.2.1. Isolation of Primary Hepatocytes

The isolation procedure was performed using a two-step collagenase perfusion protocol adapted from Akie and Cooper [53]. The isolation procedure was performed with a clamped thoracic inferior vena cava (IVC) and open perfusion from the abdominal IVC to the cut hepatic portal vein. Initially, the liver was perfused with Hanks’ Balanced Salt Solution until blood was completely drained, followed by perfusion with collagenase medium (DMEM, 1 g/L glucose supplemented with 100 units/mL collagenase IV) for tissue digestion. The digested liver tissue was dissected, and the gallbladder was removed. The liver was covered with culture medium (DMEM/F-12, supplemented with 10% fetal calf serum), and hepatocytes were released into the medium by gently scraping the liver using a scalpel. Hepatocyte homogenate was filtered (70 µm pore size), and viable hepatocytes were purified using Percoll gradient centrifugation. Cells were washed once in culture medium; cell count was determined, and the cells were plated with 40,000 cells/cm^2^ in cell culture plates coated with rat tail collagen type I. Cells sat at least 3 h at 37 °C and 5% CO_2_ prior to experiments.

#### 4.2.2. Analyses of Lipid and Carbohydrate Metabolism

Isolated primary hepatocytes in culture medium were serum-starved overnight before the start of the dependent assay. Fatty acid uptake was measured by providing radiolabeled ^3^H-palmitate in Krebs-Ringer-HEPES buffer, followed by the quantification of ^3^H activity in cell lysates after 0 and 5 min of incubation as described in detail in Benninghoff et al. [54]. The fatty acid oxidation was measured using an oxidation chamber to trap released CO_2_ from palmitate oxidation activity in hepatocytes provided with ^14^C-palmitate as substrate adapted from Benninghoff et al. [54]. Cells were incubated with an oxidation assay medium, including BSA and ^14^C-palmitate for 4 h; subsequently, oxidation was stopped by the addition of 1 mol/l HCl to release CO_2_. The oxidation chamber provided connection of adjacent wells to trap released CO_2_ from hepatocyte culture in NaOH-soaked filter papers during overnight incubation, which allowed the quantification of radiolabeled CO_2_ derived from palmitate oxidation. Control experiments to identify non-mitochondrial oxidative activity were performed with the addition of 4 µmol/L etomoxir (CPT1 inhibition). De novo lipogenesis (DNL) analysis was performed as described in detail in Akie and Cooper [53]. The hepatocytes were incubated in serum-free medium supplemented with ^14^C-acetate as substrate for DNL with and without induction using 100 nmol/L insulin. DNL was assessed using the quantification of radiolabeled lipid fractions extracted from treated hepatocyte cultures. Gluconeogenesis was measured in glucose-starved cultures of primary hepatocytes. Glucose starvation was performed for 1 h, followed by treatment of the cells with 10 nM insulin or 2 mM pyruvate/2 mM lactate to either suppress or stimulate glucose production for 4 h of incubation. Culture supernatants were centrifuged at maximal centrifugal force, and glucose concentration was assessed using the Glucose (GO) assay kit (Sigma Aldrich/Merck, Darmstadt, Germany). Glycogen synthesis experiment was performed as described in detail in Hörbelt et al. [55]. In brief, incorporation of ^14^C-glucose was measured in hepatocyte cultures with and without induction by 100 nmol/L insulin, and radiolabeled glycogen was quantified after precipitation. Glycolytic activity was measured by the extracellular acidification rate (ECAR) using an adapted protocol from the Seahorse XF Glycolysis Stress Test Kit User Guide (Agilent Technologies Inc.) with the Seahorse XFe96 extracellular flux analyzer. In brief, cells were glucose-starved one hour prior to the assay. After basal measurement (non-glycolytic acidification), 25 mmol/L glucose was injected, and the ECAR response was measured with at least 4 measurement cycles of 3 min mix and 3 min measure. The glycolysis rate was then calculated as maximum ECAR response above basal ECAR. Parameters of mitochondrial function were also assessed by extracellular flux analyses using the Seahorse XFe96 extracellular flux analyzer using the Seahorse XF Cell Mito Stress Test, Real-Time ATP Rate Assay, and Cell Energy Phenotype Test protocols as described in the respective user guides (Agilent Technologies Inc.). Compound concentrations were used as follows: 1 µmol/L oligomycin, 0.5 µmol/L FCCP, 0.5 µmol/L rotenone, and 0.5 µmol/L antimycin A. Fuel oxidation analysis was performed with additional injection of inhibitors before Mito Stress Test protocol. A combination of two inhibitors was used for each assay to specifically analyze the oxidation of glucose, glutamine, or long-chain fatty acids, respectively. Inhibitor concentrations were used as follows: 2 µmol/L UK5099, 3 µmol/BPTES, and 4 µmol/L etomoxir (all compounds: Sigma Aldrich/Merck, Darmstadt, Germany).

### 4.3. Analyses in Isolated Mitochondria

#### Analyses of Mitochondrial Respiratory Dynamics

Liver mitochondrial fractions were isolated from freshly dissected liver biopsies using a simplified differential centrifugation protocol adapted from the corresponding Application Note on ‘Analyzing Microgram Quantities of Isolated Mitochondria in the Agilent Seahorse XFe/XF24 Analyzer’ (Agilent Technologies Germany). Liver tissue (1 g) was minced in mitochondrial isolation buffer without BSA. The tissue was transferred to 10 mL fresh isolation buffer and homogenized using a drill-driven teflon pistil with a Dounce homogenizer. The homogenate was centrifuged 20 min at 600× *g*. The lipid layer was removed, and the supernatant was transferred to a fresh tube for 11,000× *g* centrifugation for 20 min. Analysis of mitochondrial respiration was performed using the pellet from 11,000× *g* centrifugation, which was resuspended in 1 mL isolation buffer (‘11,000× *g* fraction’), and the protein concentration was determined using the Bradford protein assay dye reagent (BioRad, Germany). We subjected 11,000× *g* fractions containing enriched mitochondria to extracellular flux analysis using the Seahorse XFe24 device to perform electron flow and coupling experiments as described in the manufacturer’s application note (Agilent Technologies, Inc., 2016, 5991-7145EN). In brief, 5 µg total proteins from 11,000× *g* fraction were used per well. Substrates were used at 10 mmol/L pyruvate, 5 mmol/L malate, and 10 mmol/L succinate. Compound concentrations were used as follows: 4 mmol/L ADP, 2.5 µg/mL oligomycin, 4 µmol/L FCCP, 4 µmol antimycin A, 10mM malonate, 2 µmol/L rotenone, and 100 µmol/L TMPD combined with 10 mmol/L ascorbate (all compounds purchased from Sigma Aldrich/Merck, Darmstadt, Germany). The assays were performed with coupled (FCCP addition) or uncoupled mitochondria, depending on the assay procedure, in presence of complex I-specific and/or complex II-specific substrates pyruvate/malate and succinate in the assay medium.

### 4.4. Molecular Analyses

#### 4.4.1. Proteome Analyses

Two sets of analyses were performed to obtain the proteome from mitochondria and from the entire hepatocytes. For the mitochondrial proteome, mitochondria were purified with differential isopycnic, saccharose density gradient centrifugation as described in detail in Hartwig et al. [56]. For the hepatocyte proteome, hepatocytes were isolated (Section 2.2.1) and cultured overnight in serum- and phenol-free medium. The cells were washed twice with Hanks’ Balanced Salt Solution supplemented with 1.26 mmol/L CaCl_2_. Cells were scraped from the growth area, and dry pellets were subjected to mass spectrometry. Fractions of purified mitochondria as well as hepatocyte pellets were lysed in loading buffer and subjected to 10% SDS polyacrylamide gel electrophoresis. Coomassie blue-stained protein bands were excised and digested in gel. The protein-containing gel slices were washed with 25 mmol/L ammonium bicarbonate and 25 mmol/L ammonium bicarbonate in 50% acetonitrile (*v*/*v*), successively. The proteins were reduced using 65 mmol/L dithiothreitol at 50 °C with 350× *g* for 15 min, followed by alkylation with 216 mmol/L iodoacetamide at room temperature for 15 min in the dark. The washing step was repeated as described above, and the gel slices were incubated in 100% acetonitrile for shrinking. Digestion was performed overnight at 37 °C in digestion buffer. The elution of peptides was performed with 1% trifluoroacetic acid (*v*/*v*), followed by 0.1% trifluoroacetic acid in 90% acetonitrile (*v*/*v*). The eluted peptides were lyophilized. The peptides were reconstituted for mass spectrometry analysis in 1% trifluoroacetic acid (*v*/*v*), including index retention time peptides (Biognosys, Schlieren, Switzerland). Analyses of the peptides were then performed by LC-MS/MS in a label-free proteome analysis approach with an Ultimate 3000 separation liquid chromatography system combined with an EASY-spray ion source and Orbitrap Fusion Lumos Tribrid mass spectrometer (Thermofisher Scientific, Germany). The peptides were trapped on an Acclaim PepMap C18-LC-column (ID: 75 μm, 2 cm length; Thermofisher Scientific) and separated via EASY-Spray C18 column (ES802; ID: 75 μm, 25 cm length; Thermofisher Scientific). Each LC-MS run lasted 150 min, and MS data were acquired with both data-dependent (DDA) and data-independent (DIA, 34 windows) MS/MS scan approaches. The DDA runs were analyzed using Proteome Discoverer 2.5 software (Thermofisher Scientific) and Sequest HT search (trypsin digestion, max. 2 miscleavages, 5–144 peptide length, max. 10 peptides per spectrum, carbamidomethylation as static and N-terminal acetylation/methionine oxidation as dynamic modifications) against the SwissProt FASTA database (Mus musculus (TaxID = 10090, version 2021-07). The percolator node-based peptide-spectrum match (PSM) analysis was restricted to q-values with 0.01 (strict) and 0.05 (relaxed) false discovery rate (FDR). The proteins were filtered using parsimony principle set to 0.01/0.05 (strict/relaxed) FDRs. For quantification, the DIA runs were analyzed via Spectronaut^TM^ Pulsar 14.4 software (Biognosys, Switzerland) set to standard parameter settings and using a self-performed spectral library based on DDA runs. For retention time alignment, the secretomes were spiked with the indexed retention time standard. For mitochondrial proteome as well as hepatocyte proteome, six animals per group were analyzed, and the mass spectrometry data are available at the ProteomeXchange Consortium via the PRIDE partner repository [57], with the dataset identifiers PXD032211 and PXD032214.

#### 4.4.2. Gene Expression and Trancriptome Analysis

Genome-wide expression analyses were performed with RNA from isolated primary hepatocytes using MTA arrays (Thermo Fisher Scientific, Germany) as described [58,59]. Datasets are available under NCBI GEO https://www.ncbi.nlm.nih.gov/geo/dataset, (accessed on 29 May 2022) accession numbers GSE 132298 and GSE198173. The gene expression was examined in the total RNA extracted from cultured primary murine hepatocytes by qPCR with gene-specific hybridization probes PGC-1a (Mm01247293), Tfam (Mm00447485), UcpCP2 (Mm00440940), Nrf1 (Mm00447996), and 18S (4310893E)) (ThermoFisher Scientific, Darmstadt, Germany), as described [60,61].

#### 4.4.3. Mitochondrial Copy Number

A relative copy number of the mitochondrial DNA (mtDNA) was calculated as the number of mtDNA molecules in relation to nDNA molecules by real-time qPCR expression analysis of 5 ng DNA for a mitochondria-encoded NADH dehydrogenase subunit 1 (Mt-nd1) and the nuclear-encoded single copy gene for lipoprotein lipase (Lpl), normalized to 18S rRNA. Primer sequences: Mt-nd1—forward: CTACAACCATTTGCAGACGC/–reverse: GGAACTCATAGACTTAATGC/probe: CCAATACGCCCTTTAACAACCTC; Lpl—forward: GGTTTGGATCCAGCTGGGCC/–reverse: GATTCCAATACTTCGACCAGG/probe: CTTTGAGTATGCAGAAGCCC; Eukaryotic 18S rRNA-endogenous control (VIC™/MGB-probe) (Thermo Fischer Scientific, Germany) [27].

### 4.5. Enzyme Assays

Succinate dehydrogenase and catalase enzyme activity were measured in isolated mitochondria as previously described in detail [56]. Cytochrome c Oxidase Assay Kit and Citrate Synthase Assay Kit (Sigma-Aldrich/MERCK, Darmstadt, Germany) were used for determination of enzyme activities from 10 µg or 300 µg total protein of 11,000× *g* liver fractions, respectively, according to the manufacturer’s protocols. Membrane integrities to assess the quality of mitochondria preparations were measured with the Cytochrome c Oxidase Assay Kit (Sigma-Aldrich/MERCK, Darmstadt, Germany), according to the manufacturer’s protocol. NAD+, NADH, and FAD+ abundance were also measured in 11,000× *g* liver fractions, with 10 µg total protein from 11,000× *g* fractions used with the NAD+/NADH-Glo Assay (Promega, Germany) and 0.4 µg total protein from 11,000× *g* fractions used with the Flavin Adenine Dinucleotide (FAD+) Assay Kit (Abcam, Germany), according to the provided protocols from the manufacturers. A β-Hydroxybutyrat Assay Kit (Sigma-Aldrich/MERCK, Darmstadt, Germany) was used to measure the abundance of β-hydroxybutyrat in liver tissue, according to the manufacturer’s protocol.

### 4.6. Data Analyses

Wave 2.6.0 (Agilent Technologies, Santa Clara, CA, USA), Proteome Discoverer 2.5 (Thermofisher Scientific Darmstadt, Germany), Spectronaut™ Pulsar 14.4 (Biognosys, Zürich, Switzerland), Transcriptome Analysis ConsoleTM v4.01 (ThermoFisher, Darmstadt, Germany), and knowledge-based transcriptome analyses were performed with IPA (QIAGEN Inc., Venlo, The Netherlands, www.qiagenbioinformatics.com/products/ingenuity-pathway-analysis (accessed on 29 May 2022)). We used 1.3-fold differences, *p*-value < 0.05.

### 4.7. Statistics

Statistical data analyses were performed using the GraphPad Prism software, version 9.1.2 (GraphPad Software Inc., La Jolla, CA, USA). Statistically significant differences were analyzed by two-way ANOVA, followed by Tukey’s multiple comparisons or the Mann–Whitney test, as indicated in the figure legends. Data are presented as means with the 95% confidence interval (CI), unless otherwise stated.

## 5. Conclusions

In conclusion, in early stages of MAFLD, increased lipogenic activity mediated by selective hepatic insulin resistance and the activation of SREBP-1c lead to intrahepatic accumulation of lipids. Consequently, hepatic metabolic activity adapts towards substrate supply for the synthesis of lipids, which simultaneously induces a limitation of substrates for oxidative phosphorylation to fulfill the energy demand of the cell. The metabolic balance of the cell is disturbed, and a paradox state of starvation under excess occurs. In this study, increased activity of complex II-driven mitochondrial respiration represented a rescue mechanism of the cell to use alternate substrates like glutamine-derived α-ketoglutarate to provide electron flow to maintain oxidative phosphorylation. When this state of metabolic imbalance persisted, disease pathogenesis progressed and led to decreased mitochondrial function and mitochondrial damage.

## Data Availability

Transcriptome datasets are available under NCBI GEO https://www.ncbi.nlm.nih.gov/geo/dataset (accessed on 29 May 2022) accession numbers GSE 132298 and GSE198173. Mitochondrial proteome and hepatocyte proteome data are available at the ProteomeXchange Consortium via the PRIDE partner repository, with the dataset identifier PXD032211 and PXD032214.

## Supplementary Materials

The following supporting information can be downloaded at: https://www.mdpi.com/article/10.3390/ijms23126873/s1 [62].
