# Supplementary material for: Adaptation of Oxidative Phosphorylation Machinery Compensates for Hepatic Lipotoxicity in Early Stages of MAFLD
Source: Int J Mol Sci. 2022 Jun 20;23(12):6873. doi: 10.3390/ijms23126873 (PMC9224893; doi:10.3390/ijms23126873)
Supplement: Supplementary file 1 [file ijms-23-06873-s001.zip › Supplement_table transcriptom final.pdf]

Data were analysed using the Core expression routine
https://www.qiagenbioinformatics.com/products/ingenuity-pathway-analysis
Krämer A, Green J, Pollard J, Jr, Tugendreich S. Causal analysis approaches in Ingenuity Pathway Analysis. *Bioinforma.* 2014;**30**:523–530.

IPA Settings for analyses of hepatocyte transcripts (setA) and miRNA (set B)

Analysis Settings	
Cutoff before duplicate resolution	true
Resolve duplicate on	Expression logfoldchange
Color using	Expression logfoldchange
Cutoff for Expression foldchange	down regulated -1.3 and up regulated 1.3
Cutoff for Expression pvalue	0.05
Reference set	Ingenuity Knowledge Base (Genes Only)
Relationship to include	Direct and Indirect
Includes Endogenous Chemicals	Yes

Filter Summary
Consider only molecules and/or relationships where
(confidence = High (predicted) OR Experimentally Observed) AND

(mol. types = biologic drug OR canonical pathway OR chemical - endogenous mammalian OR chemical - endogenous non-mammalian OR chemical - kinase inhibitor OR chemical - other OR chemical - protease inhibitor OR chemical drug OR chemical reagent OR chemical toxicant OR complex OR cytokine OR disease OR enzyme OR function OR G-protein coupled receptor OR group OR growth factor OR ion channel OR kinase OR ligand-dependent nuclear receptor OR mature microRNA OR microRNA OR other OR peptidase OR phosphatase OR transcription regulator OR translation regulator OR transmembrane receptor OR transporter)

Datasets show analyses ready molecules for IPA for analyses (Spring Relase 2021)

Analyses set A: hepatocyte derived transcripts

ID	Symbol	Expr Fold Cha	Expr p-value
TC0700001119.mm.1	ABHD2	3,24	9,90E-03
TC1800000794.mm.1	ACAA2	-2,78	3,84E-03
TC1100001276.mm.1	ACACA	-2,08	1,55E-02
TC1700001458.mm.1	ACAT2	1,88	2,35E-02
TC0300001413.mm.1	Adh6-ps1	-1,53	2,43E-02
TC1100001289.mm.1	Akirin1-ps	1,34	1,54E-02
TC1200000057.mm.1	APOB	1,79	3,49E-02
TC1400000862.mm.1	ARL11	1,39	8,20E-03
TC1300001298.mm.1	ARL15	-1,33	2,62E-02
TC0500003018.mm.1	ASPHD2	-1,45	3,05E-02
TC1500001568.mm.1	ATAD2	-1,34	2,10E-02
TC1500001068.mm.1	ATG101	-1,53	4,99E-03
TC1000001543.mm.1	AVIL	-1,44	2,36E-02
TC1400000853.mm.1	C1QTNF9B	-1,30	2,63E-02
TC1500000014.mm.1	C6	-1,83	3,34E-02
TC1100002158.mm.1	CAMK2B	1,44	1,81E-03
TC0700002502.mm.1	CBLC	1,36	1,04E-02
TSUnmapped00000018.m	CCDC169	-1,44	1,78E-02
TC0700002912.mm.1	Cd9-ps	1,45	4,60E-03
TC0X00002320.mm.1	Cdr1	-1,34	2,93E-02
TC0700002635.mm.1	CEACAM1	1,62	2,64E-02
TC1400002101.mm.1	CIDEB	1,64	1,78E-02
TC0800000984.mm.1	Cks1brt	-1,37	4,25E-02
TC1600001419.mm.1	CLDN1	2,54	3,52E-02
TC0X00003226.mm.1	Cldn34b2 (inc	1,31	2,34E-02
TC0100000572.mm.1	CPS1	3,56	1,77E-02
TC1900000008.mm.1	CPT1A	1,57	1,88E-02
TC1500000852.mm.1	CRELD2	-1,58	3,48E-02
TC0300000902.mm.1	CTSK	-1,32	2,38E-02
TC0100003029.mm.1	CXCR4	1,33	1,92E-02
TC1500001957.mm.1	Cyp2d26	2,10	4,15E-03
TC0400002151.mm.1	CYP7A1	-1,59	7,42E-03
TC0300001689.mm.1	CYP7B1	-1,80	3,23E-02
TC1400000891.mm.1	Defb43	1,43	8,50E-03
TC1400002213.mm.1	Defb48	1,38	1,91E-02
TC0900002720.mm.1	DPPA5	-1,36	1,23E-02
TC1700001067.mm.1	Dreh	-1,34	4,15E-02
TC1000002199.mm.1	Dux	-1,63	8,80E-03
TC0900002038.mm.1	EI24	1,47	2,20E-02
TC1300000072.mm.1	ERO1B	-1,41	2,51E-02
TC0700000388.mm.1	ETHE1	1,60	4,29E-02
TC1400001724.mm.1	Fam25c	-1,36	3,85E-02
TC0600000227.mm.1	FAM71F2	-1,65	4,83E-02
TC0300000967.mm.1	FMO5	1,78	1,79E-02
TC1100001654.mm.1	G6PC1	-2,36	2,29E-02
TC1100001622.mm.1	GAST	1,46	3,25E-02
TC1100003466.mm.1	GDPD1	-1,34	1,79E-02
TC0300000852.mm.1	Gm12854/S1	2,33	1,99E-02
TC1400000963.mm.1	GNRH1	-1,33	3,37E-02
TC0500000987.mm.1	GPAT3	-1,41	1,22E-02
TC0900002719.mm.1	GSTA5	1,38	4,78E-02
TC0600003374.mm.1	GYS2	-1,87	5,48E-03
TC0X00000120.mm.1	H2ap	1,48	9,65E-03
TC0300000930.mm.1	H2BC11	-1,37	2,42E-03
TC1300000191.mm.1	H2BC12	1,45	2,97E-02
TC0X00000987.mm.1	H3f3a-ps1	-1,50	1,59E-02
TC1600002063.mm.1	H3f3a-ps2	-1,48	1,52E-02
TC1000001133.mm.1	HAL	1,78	4,30E-02
TC1900000560.mm.1	HELLS	-1,39	3,39E-02
TC1600001496.mm.1	Hmgb1-ps6	-1,35	4,13E-02
TC0500000230.mm.1	Hspe1-ps3	1,32	4,11E-02
TC1200001663.mm.1	IFRD1	-1,66	3,48E-02
TC1200002572.mm.1	Ighv5-17	1,44	1,15E-02

Analyses set B: hepatocyte derived miRNAs

ID	Symbol	Expr Fold Cha	Expr p-value
TC0800001406.mm.1	Mir7078	-2,84	1,10E-02
TC0600003113.mm.1	mir-8	-2,27	1,35E-02
TC0900001314.mm.1	Mir2136	-2,20	2,13E-02
TC1800000626.mm.1	Mir6983	-2,10	7,67E-03
TC0400002710.mm.1	mir-32	-2,05	1,70E-02
TC1200002373.mm.1	mir-299	-1,92	3,23E-02
TC0200001439.mm.1	Mir7221	-1,90	6,39E-03
TC0600002009.mm.1	mir-29	-1,85	1,89E-03
TC1000000897.mm.1	Mir6913	-1,78	7,04E-03
TC1400002294.mm.1	Mir6539	-1,76	2,10E-02
TC0800001278.mm.1	mir-140	-1,66	1,52E-03
TC0400000355.mm.1	Mir3094	-1,61	1,94E-02
TC0X00002216.mm.1	Mir6384	-1,51	2,78E-02
TC1000001865.mm.1	Mir7663	-1,46	1,22E-02
TC1200002444.mm.1	Mir6941	-1,42	2,04E-03
TC0700004590.mm.1	Mir7064	-1,39	1,50E-02
TC1500000783.mm.1	Mir6392	-1,37	1,18E-03
TC0X00002779.mm.1	mir-95	-1,36	2,32E-02
TC0700001433.mm.1	mir-139	-1,36	6,21E-03
TC1100001544.mm.1	Mir6927	-1,35	3,07E-02
TC1000000575.mm.1	Mir5108	-1,34	3,86E-02
TC0200000111.mm.1	mir-467	-1,32	1,25E-02
TC1300002100.mm.1	mir-7	-1,30	1,83E-02
TC0100001558.mm.1	Mir6348	1,30	1,01E-02
TC1000000880.mm.1	mir-5615	1,31	7,64E-03
TC1700000674.mm.1	Mir6973a	1,32	1,38E-02
TC1800000565.mm.1	Mir1258	1,32	2,62E-02
TC0200004528.mm.1	Mir210	1,32	3,04E-02
TC0200002786.mm.1	Mir3091	1,32	8,05E-03
TC0800002464.mm.1	Mir7068	1,34	5,97E-03
TC0900000076.mm.1	Mir6237	1,34	2,40E-02
TC1800001079.mm.1	mir-187	1,36	3,42E-04
TC0X00002370.mm.1	mir-506	1,36	3,34E-02
TC0200004139.mm.1	Mir7000	1,36	1,50E-02
TC1600001505.mm.1	Mir1946a	1,36	1,06E-02
TC0800000951.mm.1	mir-23	1,37	3,93E-02
TC0700002618.mm.1	Mir7047	1,39	2,71E-02
TC1800000106.mm.1	Mir1948	1,39	2,64E-02
TC0100003622.mm.1	mir-350	1,40	2,70E-02
TC1100001187.mm.1	mir-193	1,41	6,61E-03
TC1500000168.mm.1	Mir7212	1,42	1,55E-03
TC1400000997.mm.1	mir-320	1,50	1,61E-02
TC0300001577.mm.1	mir-186	1,50	3,64E-02
TC0200000712.mm.1	mir-181	1,50	7,11E-03
TSUnmapped000001:	Mir5118	1,52	8,01E-03
TC1400002114.mm.1	Mir6949	1,53	6,66E-03
TSUnmapped000001(	Mir7660	1,56	3,68E-03
TC1200000237.mm.1	Mir6937	1,57	1,08E-03
TC1100000870.mm.1	Mir6406	1,57	4,72E-02
TC0500003235.mm.1	Mir7647	1,58	1,47E-02
TC1100001491.mm.1	mir-196	1,58	5,78E-03
TC0800000967.mm.1	Mir7069	1,58	9,42E-04
TC0X00000671.mm.1	Mir2137	1,59	4,25E-02
TC0800002873.mm.1	Mir7075	1,60	3,41E-03
TC0400001466.mm.1	Mir7119	1,64	3,11E-03
TC1000000849.mm.1	Mir6911	1,65	2,40E-02
TC1500001122.mm.1	mir-615	1,67	2,46E-03
TC1200001172.mm.1	mir-379	1,67	4,31E-03
TC1100002870.mm.1	Mir6921	1,67	7,02E-04
TC0900001852.mm.1	Mir7081	1,67	1,49E-02
TC1200001143.mm.1	mir-341	1,69	2,87E-02
TC1200001178.mm.1	mir-329	1,69	1,52E-02
TC1500000819.mm.1	let-7	1,70	2,24E-02

TC0600003579.mm.1	Igkv12-41	-1,52	3,32E-02
TC0600003581.mm.1	Igkv5-39	1,37	2,56E-02
TC0600003587.mm.1	Igkv8-28	1,55	1,98E-02
TC0600001252.mm.1	IL17RC	1,31	5,00E-02
TC0100002969.mm.1	INSIG2	2,04	3,28E-02
TC0800001694.mm.1	IRS2	1,72	1,08E-02
TC0100002762.mm.1	KCNJ13	1,31	2,30E-02
TC1500001410.mm.1	KLF10	-1,34	4,49E-02
TC0600000822.mm.1	KRCC1	1,30	3,36E-02
TC1600002019.mm.1	KRTAP19-3	-1,53	4,73E-03
TC0100000717.mm.1	Krtap28-13	-1,80	3,23E-02
TC0700004559.mm.1	Krtap5-5	-1,34	3,63E-02
TC1600002033.mm.1	Krtap6-2	-1,94	4,57E-02
TC0200003265.mm.1	KYAT1	1,63	3,92E-02
TC0300001448.mm.1	KYAT3	-1,73	3,15E-02
TC0800000123.mm.1	LAMP1	2,17	2,26E-02
TC0X00002085.mm.1	LAMP2	2,27	1,45E-02
TC1500000819.mm.1	let-7	1,70	2,24E-02
TC1100003314.mm.1	LGALS9B	2,37	4,32E-02
TC1900001419.mm.1	LIPA	2,03	1,72E-02
TC1200001662.mm.1	LSMEM1	-1,85	5,98E-03
TC0X00003231.mm.1	Magea3 (inclu	-1,37	1,34E-02
TC1300000021.mm.1	Marcksl1-ps4	-1,39	1,69E-02
TC0100003026.mm.1	MCM6	-1,60	4,40E-02
TC1500000380.mm.1	MED30	-1,32	3,71E-02
TC0900001334.mm.1	mir-135	1,79	2,07E-02
TC0700001433.mm.1	mir-139	-1,36	6,21E-03
TC0800001278.mm.1	mir-140	-1,66	1,52E-03
TC1200001174.mm.1	mir-154	1,96	3,72E-02
TC0200000712.mm.1	mir-181	1,50	7,11E-03
TC0300001577.mm.1	mir-186	1,50	3,64E-02
TC1800001079.mm.1	mir-187	1,36	3,42E-04
TC1100001187.mm.1	mir-193	1,41	6,61E-03
TC1100001491.mm.1	mir-196	1,58	5,78E-03
TC0800000951.mm.1	mir-23	1,37	3,93E-02
TC0600002009.mm.1	mir-29	-1,85	1,89E-03
TC1200002373.mm.1	mir-299	-1,92	3,23E-02
TC0400002710.mm.1	mir-32	-2,05	1,70E-02
TC1400000997.mm.1	mir-320	1,50	1,61E-02
TC1200001178.mm.1	mir-329	1,69	1,52E-02
TC1200001143.mm.1	mir-341	1,69	2,87E-02
TC0100003622.mm.1	mir-350	1,40	2,70E-02
TC1200001172.mm.1	mir-379	1,67	4,31E-03
TC0200000111.mm.1	mir-467	-1,32	1,25E-02
TC0X00002370.mm.1	mir-506	1,36	3,34E-02
TC1000000880.mm.1	mir-5615	1,31	7,64E-03
TC1500001122.mm.1	mir-615	1,67	2,46E-03
TC1300002100.mm.1	mir-7	-1,30	1,83E-02
TC0600003113.mm.1	mir-8	-2,27	1,35E-02
TC0X00002779.mm.1	mir-95	-1,36	2,32E-02
TC1600001462.mm.1	Mir1195	1,77	9,71E-03
TC1800000565.mm.1	Mir1258	1,32	2,62E-02
TC1600001505.mm.1	Mir1946a	1,36	1,06E-02
TC1800000106.mm.1	Mir1948	1,39	2,64E-02
TC0900001314.mm.1	Mir2136	-2,20	2,13E-02
TC0X00000671.mm.1	Mir2137	1,59	4,25E-02
TC1100002410.mm.1	Mir216c	1,74	2,70E-02
TC0700002122.mm.1	Mir290b	1,80	1,12E-02
TC1000002730.mm.1	Mir3058	2,56	1,23E-02
TC0200002786.mm.1	Mir3091	1,32	8,05E-03
TC0400000355.mm.1	Mir3094	-1,61	1,94E-02
TC1400001229.mm.1	Mir3964	2,11	3,61E-02
TC1000000575.mm.1	Mir5108	-1,34	3,86E-02
TSUnmapped00000118.m	Mir5118	1,52	8,01E-03
TC0900000076.mm.1	Mir6237	1,34	2,40E-02
TSUnmapped00000115.m	Mir6240	3,65	2,26E-03
TC0100001558.mm.1	Mir6348	1,30	1,01E-02

TC1600002159.mm.1	Mir6964	1,71	4,84E-02
TC1400002064.mm.1	Mir6948	1,71	2,12E-02
TC1100002410.mm.1	Mir216c	1,74	2,70E-02
TC1600001462.mm.1	Mir1195	1,77	9,71E-03
TC0X00002218.mm.1	Mir717	1,79	2,68E-02
TC0900001334.mm.1	mir-135	1,79	2,07E-02
TC0700002122.mm.1	Mir290b	1,80	1,12E-02
TC1800000604.mm.1	Mir6355	1,81	7,24E-03
TC1800001223.mm.1	Mir6980	1,85	5,73E-04
TC1200001174.mm.1	mir-154	1,96	3,72E-02
TC0500000874.mm.1	Mir6415	1,97	1,96E-02
TC0800002952.mm.1	Mir7076	2,00	2,58E-04
TC1400001229.mm.1	Mir3964	2,11	3,61E-02
TC0900001862.mm.1	Mir7667	2,48	3,84E-02
TC1000002730.mm.1	Mir3058	2,56	1,23E-02
TSUnmapped000001	Mir6240	3,65	2,26E-03

TC1800000604.mm.1	Mir6355	1,81	7,24E-03
TC0X00002216.mm.1	Mir6384	-1,51	2,78E-02
TC1500000783.mm.1	Mir6392	-1,37	1,18E-03
TC1100000870.mm.1	Mir6406	1,57	4,72E-02
TC0500000874.mm.1	Mir6415	1,97	1,96E-02
TC1400002294.mm.1	Mir6539	-1,76	2,10E-02
TC1000000849.mm.1	Mir6911	1,65	2,40E-02
TC1000000897.mm.1	Mir6913	-1,78	7,04E-03
TC1100002870.mm.1	Mir6921	1,67	7,02E-04
TC1100001544.mm.1	Mir6927	-1,35	3,07E-02
TC1200000237.mm.1	Mir6937	1,57	1,08E-03
TC1200002444.mm.1	Mir6941	-1,42	2,04E-03
TC1400002064.mm.1	Mir6948	1,71	2,12E-02
TC1400002114.mm.1	Mir6949	1,53	6,66E-03
TC1600002159.mm.1	Mir6964	1,71	4,84E-02
TC1700000674.mm.1	Mir6973a	1,32	1,38E-02
TC1800001223.mm.1	Mir6980	1,85	5,73E-04
TC1800000626.mm.1	Mir6983	-2,10	7,67E-03
TC0200004139.mm.1	Mir7000	1,36	1,50E-02
TC0700002618.mm.1	Mir7047	1,39	2,71E-02
TC0700004590.mm.1	Mir7064	-1,39	1,50E-02
TC0800002464.mm.1	Mir7068	1,34	5,97E-03
TC0800000967.mm.1	Mir7069	1,58	9,42E-04
TC0800002873.mm.1	Mir7075	1,60	3,41E-03
TC0800002952.mm.1	Mir7076	2,00	2,58E-04
TC0800001406.mm.1	Mir7078	-2,84	1,10E-02
TC0900001852.mm.1	Mir7081	1,67	1,49E-02
TC0400001466.mm.1	Mir7119	1,64	3,11E-03
TC0X00002218.mm.1	Mir717	1,79	2,68E-02
TC1500000168.mm.1	Mir7212	1,42	1,55E-03
TC0200001439.mm.1	Mir7221	-1,90	6,39E-03
TC0500003235.mm.1	Mir7647	1,58	1,47E-02
TSUnmapped00000109.m	Mir7660	1,56	3,68E-03
TC1000001865.mm.1	Mir7663	-1,46	1,22E-02
TC0900001862.mm.1	Mir7667	2,48	3,84E-02
TSUnmapped00000114.m	Mir7689	-1,41	3,07E-03
TC0100003301.mm.1	MR1	1,32	2,64E-03
TC0M00000006.mm.1	MT-CO1	1,32	1,95E-03
TC0M00000005.mm.1	MT-ND2	2,38	8,60E-03
TC0M00000011.mm.1	MT-ND3	1,51	9,39E-03
TC0X00001524.mm.1	n-R5s13	1,58	3,81E-02
TC0600000722.mm.1	n-R5s162	3,74	5,25E-05
TC0300000011.mm.1	n-R5s194	1,86	2,15E-03
TC0100000354.mm.1	n-R5s211	1,90	6,21E-03
TC1600000459.mm.1	n-R5s33	2,13	5,86E-04
TC1200002099.mm.1	n-R5s64	2,00	3,72E-04
TC0100002319.mm.1	NAB1	1,31	1,65E-02
TC0600003600.mm.1	Nat8f5	-1,33	3,94E-02
TC1700001015.mm.1	NDUFA11	1,50	1,88E-02
TC1000001566.mm.1	NDUFA4L2	1,99	3,16E-02
TC0700002124.mm.1	NLRP12	1,31	3,77E-02
TC1300002740.mm.1	Nnt	1,30	4,76E-03
TC1100003688.mm.1	NR1D1	-3,13	2,34E-02
TC1000002657.mm.1	NR1H4	1,67	2,51E-02
TC0900002332.mm.1	NRG4	2,99	6,88E-03
TC0900002988.mm.1	NUDT16	1,32	2,26E-02
TC0800001097.mm.1	NUP93	-1,33	2,91E-02
TC0700004409.mm.1	OAT	-3,41	4,22E-02
TC0200004019.mm.1	Olfr1180	1,40	1,23E-02
TC0200004054.mm.1	Olfr1233	1,37	2,40E-02
TC1900000262.mm.1	Olfr1451	-1,57	4,09E-02
TC0900000156.mm.1	Olfr58 (incluc	-1,31	1,11E-02
TC0700004015.mm.1	Olfr695	1,38	2,27E-02
TC1000003181.mm.1	Olfr777/Olfr8	1,53	2,79E-02
TC0700001572.mm.1	OR2D3	1,47	4,76E-02
TC0200004077.mm.1	OR4C3	-1,55	4,03E-02
TC0600000415.mm.1	OR9A4	-1,38	5,51E-03

TC0400001390.mm.1	Oxct2a/Oxct2	-1,67	1,04E-02
TC0600003317.mm.1	Pbp2	1,59	1,55E-02
TC1900000040.mm.1	PC	-1,72	9,88E-03
TC0200002683.mm.1	PCK1	-2,61	1,49E-02
TC1400001436.mm.1	PDHB	-1,91	6,46E-03
TC1100003107.mm.1	PFN1	1,76	2,48E-02
TC1200002089.mm.1	PGF	-1,48	3,50E-02
TC1700002101.mm.1	PGK2	1,89	2,41E-03
TC1100000001.mm.1	Pisd-ps1	-1,61	3,57E-03
TC1700001348.mm.1	Pisd-ps2	-1,43	3,66E-03
TC0600003329.mm.1	PLBD1	-1,33	2,48E-02
TC1100003934.mm.1	POLG2	-1,30	1,96E-02
TC0200005386.mm.1	PPP1R3D	-1,39	1,83E-02
TC0500000809.mm.1	Prol1	2,10	2,73E-03
TC0700004346.mm.1	PRSS8	1,41	3,75E-02
TC1900001203.mm.1	PSAT1	-1,40	2,63E-02
TC1100004291.mm.1	Psme2b	1,47	1,01E-03
TC0900001410.mm.1	Qars	-1,61	2,29E-02
TC0600002978.mm.1	RASSF4	1,92	9,67E-03
TC1000003221.mm.1	RDH16	-1,70	4,49E-02
TC0600001741.mm.1	REP15	-1,79	5,26E-03
TC0X00000359.mm.1	Rhox10	1,36	2,44E-02
TC0600002206.mm.1	Rn4.5s	1,81	1,58E-02
TC0700004658.mm.1	RNF141	-1,33	2,00E-02
TSUnmapped00000181.m	RNY1	2,82	4,01E-02
TSUnmapped00000180.m	Rny3	2,05	1,86E-03
TC1200001841.mm.1	RPL10L	1,31	4,47E-02
TC0100002198.mm.1	Rpl12-ps1	1,52	7,84E-03
TC1100002300.mm.1	Rpl12-ps2	1,70	1,32E-03
TC0200000708.mm.1	Rpl15-ps4	1,47	8,49E-03
TC0100000486.mm.1	Rpl18-ps1	1,66	4,55E-02
TC1200001765.mm.1	Rpl18a-ps1	1,73	1,24E-02
TC1100000971.mm.1	Rpl23a-ps2	1,32	3,01E-02
TC0200002038.mm.1	Rpl23a-ps4	1,37	2,45E-02
TC0400003526.mm.1	Rpl28-ps3	1,70	4,09E-02
TC0X00000701.mm.1	Rpl3-ps2	1,42	3,18E-02
TC0200005315.mm.1	Rpl30-ps5	1,30	4,90E-02
TC0300000292.mm.1	Rpl31-ps15	1,56	3,94E-02
TC1900000742.mm.1	Rpl31-ps18	1,49	2,57E-02
TC1500000779.mm.1	Rpl31-ps22	1,55	4,56E-02
TC0800002043.mm.1	Rpl31-ps23	1,56	1,68E-03
TC0100000543.mm.1	Rpl31-ps24	1,42	3,82E-02
TC1700002614.mm.1	Rpl31-ps25	1,42	2,90E-02
TC0500000166.mm.1	Rpl31-ps5	1,57	2,43E-02
TC0500001420.mm.1	Rpl31-ps6	1,37	2,26E-02
TC0600002210.mm.1	Rpl31-ps7	1,44	3,81E-02
TC0X00002057.mm.1	RPL39	1,41	2,56E-02
TC1500001107.mm.1	Rpl39-ps	1,62	1,60E-02
TC0X00000606.mm.1	Rpl7a-ps13	1,41	1,54E-02
TC1100003400.mm.1	Rpl9-ps1	1,46	3,03E-03
TC1100000607.mm.1	Rpl9-ps3	1,31	3,66E-02
TC1700002025.mm.1	Rpl9-ps9	1,36	1,74E-02
TC0300000845.mm.1	Rplp0-ps1	1,45	2,69E-02
TC0800001620.mm.1	Rprl3	-1,53	4,52E-02
TC1100001719.mm.1	RPRML	-1,34	1,45E-02
TC0500003620.mm.1	Rps10-ps4	1,45	1,27E-02
TC0900002411.mm.1	Rps11-ps1	1,53	2,29E-02
TC0X00001511.mm.1	Rps12-ps7	1,33	1,23E-02
TC0500002904.mm.1	Rps15a-ps5	1,75	6,09E-03
TC0500002939.mm.1	Rps19-ps8	1,40	2,79E-03
TC0600002670.mm.1	Rps19-ps9	1,35	2,82E-02
TC1100000610.mm.1	Rps2-ps4	1,49	1,65E-02
TC1300001017.mm.1	RPS23	1,38	3,18E-02
TC0800001611.mm.1	Rps23-ps2	1,54	2,93E-02
TC0100000516.mm.1	Rps27-ps1	1,46	4,17E-02
TC0400002749.mm.1	Rpsa-ps11	1,65	3,83E-03
TC1100000285.mm.1	Rpsa-ps5	1,40	1,51E-03

TC0700000826.mm.1	RRAS	2,41	3,02E-02
TC0300000809.mm.1	S100A6	1,54	7,58E-03
TC0300000811.mm.1	S100A8	1,36	1,15E-02
TC0700003039.mm.1	Saa3	-1,81	2,78E-02
TSUnmapped00000007.m	SAMD15	1,32	3,94E-03
TC0600003137.mm.1	Scarna10	-1,85	4,19E-02
TC0700002025.mm.1	Scgb1c1	-1,37	3,89E-02
TC0100003528.mm.1	SDHC	1,61	1,10E-02
TC0900002271.mm.1	SDHD	1,50	9,73E-03
TC1500000002.mm.1	SELENOP	1,43	4,78E-02
TC1200001044.mm.1	SERPINA3	1,54	3,96E-02
TC1200002290.mm.1	Serpina3d-ps	1,49	1,60E-02
TC1200002291.mm.1	Serpina3e-ps	1,61	5,92E-03
TC1200002526.mm.1	Serpina3h	1,31	3,35E-03
TC1100004285.mm.1	SFI1	-1,74	3,94E-02
TC0200001524.mm.1	SLC1A2	-1,87	6,42E-04
TC1700001450.mm.1	SLC22A1	-2,14	3,34E-02
TC1100003836.mm.1	SLC25A39	1,36	4,11E-02
TC0100002288.mm.1	SLC39A10	-1,30	8,39E-03
TC1600001019.mm.1	SLC5A3	-1,34	1,92E-02
TC0800000482.mm.1	SLC7A2	2,47	3,09E-03
TC0900001297.mm.1	SLCO2A1	2,00	4,66E-02
TC0500003714.mm.1	Smok2b (inclu	-1,81	3,80E-02
TC0100000521.mm.1	Snora41	-2,22	1,39E-02
TC1100003289.mm.1	Snord42a	2,56	2,91E-02
TC0500001025.mm.1	SPP1	1,60	3,25E-02
TC0Y00000240.mm.1	Sry	1,42	1,98E-02
TC0X00000122.mm.1	SYTL5	2,03	4,72E-02
TC1000000203.mm.1	Taar7b (inclu	-1,56	4,94E-02
TC0600002427.mm.1	TACSTD2	1,36	2,87E-02
TC1100001756.mm.1	TANC2	-1,34	3,08E-02
TC0600000463.mm.1	TAS2R41	-1,30	4,56E-02
TC1200001054.mm.1	Tcl1b4 (incluc	-1,58	3,10E-02
TC0900002119.mm.1	TECTA	-1,32	6,36E-03
TC1600000440.mm.1	TM4SF19	-1,42	3,09E-02
TC1600002152.mm.1	TMPRSS2	1,62	4,06E-02
TC1400000726.mm.1	Traj57	1,49	2,80E-02
TC1400002826.mm.1	Trav4-4-dv10	-1,46	2,23E-02
TC1400002804.mm.1	Trav6d-7	-1,80	2,06E-02
TC1400002801.mm.1	Trav9d-4	-1,41	1,94E-02
TC0200004896.mm.1	TRIB3	-1,31	3,15E-02
TC1000001068.mm.1	Tyms-ps	-1,38	5,51E-03
TC1000003216.mm.1	UBE2G2	-1,40	4,23E-02
TC0100000682.mm.1	UTP14C	-1,39	4,38E-02
TC1900000415.mm.1	VLDLR	-2,01	1,50E-02
TC0600000694.mm.1	Vmn1r19	1,43	1,79E-03
TC1300000231.mm.1	Vmn1r192 (ir	-1,77	2,27E-03
TC0700003665.mm.1	Vmn2r-ps65	1,33	1,59E-02
TC1700001536.mm.1	Vmn2r106	1,31	3,26E-02
TC1000001675.mm.1	ZBTB2	-1,37	5,16E-03
TC1300002228.mm.1	Zfp640	-2,05	3,69E-02
TC1100003597.mm.1	Zfp652os	1,50	9,74E-04
TC0200005514.mm.1	ZHX3	1,35	7,26E-03
TC0700000368.mm.1	ZNF180	-1,33	1,32E-03
TC0400003995.mm.1	ZNF616	-1,42	2,19E-02

Upstream Regulators in hepatocytes transcriptomes deduced from datasets. Top 50 hits with highest p-value of enrichment are indicated.

Analyses set A: hepatocyte derived transcripts

Upstream Regulator	upstream re	Molecule Type	Predicted Activation State	Activation z-score	p-value of overlap	Target molecules in dataset
AGO2		translation regulator		0,36	1,97E-18	CXCR4,let-7,mir-135,mir-139,mir-140,mir-154,mir-181,mir-186,mir-187,mir-193,mir-23,mir-29,mir-320,mir-329,mir-379,mir-467,mir-7,mir-8,MT-CO1,MT-ND2
PPARA		ligand-dependent nuclear receptor		0,29	4,63E-10	ACAA2,ACACA,ACAT2,C6,CEACAM1,CPS1,CPT1A,CYP7A1,CYP7B1,ETHE1,G6PC1,INSIG2,KYAT1,KYAT3,let-7,mir-193,mir-8,NR1D1,PC,PCK1,S100A8
NR0B2		ligand-dependent nuclear receptor		-0,63	6,81E-10	ACACA,CPT1A,CYP7A1,CYP7B1,G6PC1,GSTA5,mir-154,mir-329,mir-379,NR1H4,PCK1,SPP1
1,4-bis[2-(3,5-dichloropyridyloxy)]benzen		chemical toxicant		1,60	4,51E-09	ACACA,CPT1A,CYP7A1,CYP7B1,EI24,G6PC1,GSTA5,INSIG2,MCM6,PCK1,TMPRSS2
PPARGC1A		transcription regulator		0,28	6,21E-09	ACACA,CIDEB,CPT1A,CYP7A1,G6PC1,INSIG2,IRS2,KYAT1,KYAT3,LGALS9B,LIPA,MCM6,MT-CO1,NR1H4,PCK1,Saa3,SDHD,SERPINA3,TRIB3
LGR5		transmembrane receptor	Inhibited	-2,45	1,45E-08	HAL,OAT,PLBD1,RDH16,SLC1A2,SLC22A1
ALOX5		enzyme		-0,79	1,90E-08	CTSK,let-7,mir-140,mir-181,mir-186,mir-193,mir-196,mir-23,mir-29,mir-95
rimonabant		chemical drug		-0,64	3,86E-08	mir-139,mir-154,mir-379,mir-467,mir-8,Mir216c,Mir6937,Mir6980,Mir7047,Mir7081,Mir7221
PARN		enzyme	Activated	2,20	4,49E-08	let-7,mir-181,mir-193,mir-379,RNY1
SSB		enzyme		0,28	4,90E-08	let-7,mir-135,mir-139,mir-140,mir-186,mir-193,mir-29,mir-320,mir-95
TO-901317		chemical reagent		0,25	1,01E-07	ACACA,CPT1A,CYP7A1,CYP7B1,FMO5,G6PC1,GSTA5,MCM6,NR1D1,PCK1,RDH16,SERPINA3,VLDLR
D-glucose		chemical - endogenous mammalian		-0,32	1,79E-07	ACAA2,ACACA,APOB,CXCR4,CYP7A1,ERO1B,G6PC1,IRS2,KLF10,mir-154,mir-29,mir-329,NR1H4,OAT,PC,PCK1,RDH16,S100A8,Saa3,SELENOP,SPP1,TACSTD2
INSR		kinase		0,31	2,01E-07	ACAA2,CPT1A,CXCR4,HELLS,INSIG2,KLF10,let-7,mir-139,mir-154,mir-196,mir-299,mir-329,mir-341,mir-379,mir-467,MT-CO1,PCK1,PDHB,SELENOP
CEBPA		transcription regulator		0,36	2,72E-07	ACACA,APOB,CPS1,CPT1A,CTSK,CXCR4,CYP7A1,G6PC1,IRS2,mir-181,MT-CO1,NR1H4,PCK1,S100A8,Saa3,SPP1,VLDLR
ciprofibrate		chemical drug		0,63	3,97E-07	APOB,CEACAM1,CPS1,CYP7A1,CYP7B1,G6PC1,HAL,OAT,Saa3,SLCO2A1
RNA polymerase iii		complex		-0,45	5,16E-07	mir-154,mir-181,mir-506,mir-615,mir-7
tamoxifen		chemical drug		1,92	5,44E-07	ACACA,CXCR4,CYP7A1,GSTA5,mir-181,mir-23,mir-8,MT-CO1,PFN1,SERPINA3,SLC1A2,SLC5A3,SLC7A2,SPP1
SREBF1		transcription regulator		1,76	5,98E-07	ACACA,CYP7A1,CYP7B1,ETHE1,G6PC1,IRS2,MT-CO1,MT-ND2,MT-ND3,OAT,PCK1,SELENOP,SERPINA3
lipopolysaccharide		chemical drug		1,52	6,53E-07	ACAA2,APOB,CEACAM1,CLDN1,CPT1A,CRELD2,CTSK,CXCR4,CYP7A1,CYP7B1,GNRH1,HAL,IFRD1,KLF10,LAMP1,let-7,LIPA,mir-154,mir-193,mir-320,mir-379,mir-467,mir-506,mir-8,mir-95,MT-CO1,NR1H4,NUP93,PC,S100A8,Saa3,SELENOP,SERPINA3,SLC1A2,SLC22A1,SLC39A10,SLC7A2,SLCO2A1,SPP1,TANC2,TRIB3,VLDLR
CTNNB1		transcription regulator		0,19	8,24E-07	ACACA,ACAT2,C6,CEACAM1,CPT1A,CYP7B1,Defb48,G6PC1,GAST,GPAT3,LIPA,NDUFA11,OAT,PCK1,PFN1,RASSF4,RPL39,RPS23,S100A8,Saa3,SDHC,SDHD,SERPINA3,SLC1A2,SPP1
RORC		ligand-dependent nuclear receptor			9,77E-07	CYP7B1,G6PC1,GSTA5,Nat8f5,PCK1,RDH16,S100A8,SLC1A2,SLC7A2,VLDLR
INS		other		-0,53	1,22E-06	ACACA,CPT1A,G6PC1,IRS2,mir-29,PCK1,RDH16,SDHC,TRIB3,VLDLR
IGF1R		transmembrane receptor		1,44	1,35E-06	IRS2,let-7,mir-139,mir-154,mir-196,mir-299,mir-329,mir-341,mir-379,mir-467,MT-CO1,MT-ND3,SPP1
PPARG		ligand-dependent nuclear receptor		-0,77	1,42E-06	ACAA2,ACACA,CPT1A,G6PC1,Gm12854/S100a11,INSIG2,IRS2,let-7,mir-29,NR1D1,PC,PCK1,PDHB,S100A8,SPP1,TRIB3,VLDLR
Smad2/3		group		1,28	1,84E-06	mir-154,mir-181,mir-320,mir-329,mir-341,mir-467,mir-7
RORA	-0,675	ligand-dependent nuclear receptor			1,89E-06	CYP7B1,G6PC1,GSTA5,Nat8f5,NR1D1,PCK1,RDH16,S100A8,Saa3,SLC1A2,VLDLR
SRD5A1		enzyme		-1,00	2,10E-06	ACACA,CLDN1,G6PC1,IRS2,PCK1
Gnasas1		other		-0,90	2,11E-06	let-7,mir-186,mir-196,mir-23,mir-7,mir-8
methapyrilene		chemical drug	Inhibited	-2,45	2,45E-06	CPS1,CPT1A,CYP7A1,GYS2,IFRD1,LGALS9B,NR1H4,PC,SELENOP
EPHB6		kinase	Activated	2,20	2,73E-06	let-7,mir-23,mir-29,mir-32,mir-506,TRIB3
LGR4		transmembrane receptor	Inhibited	-2,45	3,09E-06	HAL,OAT,PLBD1,RDH16,SLC1A2,SLC22A1
ELOVL5		enzyme		1,29	3,10E-06	ACACA,G6PC1,INSIG2,PCK1,TRIB3
acetaminophen		chemical drug		-1,63	3,43E-06	CPS1,GSTA5,IFRD1,let-7,mir-154,mir-193,mir-29,mir-467,NR1H4,SELENOP
NR1I3		ligand-dependent nuclear receptor	Activated	2,03	3,53E-06	CYP7A1,CYP7B1,EI24,G6PC1,GSTA5,INSIG2,PCK1,TMPRSS2
ethanol		chemical - endogenous mammalian		-1,49	4,70E-06	ACACA,CPT1A,CXCR4,CYP7A1,CYP7B1,DPPA5,G6PC1,GSTA5,let-7,mir-154,mir-29,mir-8,PCK1,SLC1A2,TRIB3
Insulin		group		-0,72	5,05E-06	ACACA,CYP7A1,G6PC1,GAST,GNRH1,INSIG2,IRS2,MCM6,mir-181,mir-193,mir-29,mir-615,mir-95,NR1H4,PCK1,PDHB,Saa3,SELENOP,VLDLR
YAP1		transcription regulator			5,36E-06	ACACA,ACAT2,CPT1A,CXCR4,HAL,let-7,MCM6,mir-135,mir-196,mir-29,RASSF4,RPL39,RPS23
dihydrotestosterone		chemical - endogenous mammalian		1,38	5,37E-06	ACACA,ATAD2,CPT1A,G6PC1,Gm12854/S100a11,GNRH1,IRS2,mir-32,MT-CO1,MT-ND3,PC,PCK1,PFN1,RPS23,SELENOP,SPP1,TMPRSS2,VLDLR
cocaine		chemical drug		1,23	5,48E-06	CPT1A,G6PC1,IRS2,mir-154,mir-181,mir-186,mir-379,mir-467,mir-7,SLC1A2
cholesterol		chemical - endogenous mammalian		-1,20	5,66E-06	ACACA,APOB,CPT1A,CTSK,CYP7A1,CYP7B1,GSTA5,PSAT1,S100A8,Saa3,TM4SF19
Gcg		other		1,63	8,41E-06	CPS1,ERO1B,GSTA5,HAL,OAT,SLC7A2
hydrogen peroxide		chemical - endogenous mammalian		0,42	9,07E-06	ACAT2,C1QTNF9B,CXCR4,GSTA5,HELLS,let-7,mir-181,mir-193,mir-23,mir-29,mir-7,MT-CO1,PGF,SELENOP,SPP1,TRIB3
APC		enzyme		0,19	9,93E-06	APOB,CLDN1,let-7,mir-181,mir-196,mir-379,mir-8,OAT,SPP1
BB-Cl-amidine		chemical reagent			1,10E-05	let-7,mir-23,mir-29
xylitol		chemical - endogenous mammalian			1,10E-05	G6PC1,NR1H4,PCK1
tretinoin		chemical - endogenous mammalian		1,08	1,13E-05	ABHD2,CCDC169,CEACAM1,CLDN1,CPT1A,CTSK,CXCR4,CYP7A1,GSTA5,KLF10,LAMP2,let-7,LIPA,mir-135,mir-139,mir-154,mir-181,mir-23,mir-32,mir-329,mir-379,mir-615,mir-7,Nnt,OAT,PCK1,PFN1,S100A8,SLC5A3,SLC7A2,SLCO2A1,SPP1
cisplatin		chemical drug		0,81	1,14E-05	CPT1A,EI24,G6PC1,let-7,LIPA,mir-135,mir-140,mir-181,mir-186,mir-193,mir-196,mir-23,mir-29,mir-32,mir-467,mir-7,mir-8,NR1H4,OAT,SPP1
GW 4064		chemical toxicant		0,43	1,24E-05	ACACA,CYP7A1,G6PC1,NR1H4,PCK1,TRIB3
GPD1		enzyme			1,33E-05	C6,CYP7B1,GPAT3,GSTA5,PSAT1,SERPINA3,VLDLR
SLC25A13		transporter			1,52E-05	C6,CYP7B1,GPAT3,GSTA5,PSAT1,SERPINA3,VLDLR

Analyses set B: hepatocyte derived miRNAs

Upstream Regulator	upstream re	Molecule Type	Predicted Activation State	Activation z-score	p-value of overlap	Target molecules in dataset
AGO2		translation regulator		1,02	5,04E-28	let-7,mir-135,mir-139,mir-140,mir-154,mir-181,mir-186,mir-187,mir-193,mir-23,mir-29,mir-320,mir-329,mir-379,mir-467,mir-7,mir-8
rimonabant		chemical drug		-0,64	3,11E-16	mir-139,mir-154,mir-379,mir-467,mir-8,Mir216c,Mir6937,Mir6980,Mir7047,Mir7081,Mir7221
SSB		enzyme		0,28	1,23E-14	let-7,mir-135,mir-139,mir-140,mir-186,mir-193,mir-29,mir-320,mir-95
ALOX5		enzyme		-0,49	6,93E-14	let-7,mir-140,mir-181,mir-186,mir-193,mir-196,mir-23,mir-29,mir-95
Smad2/3		group		1,28	1,63E-11	mir-154,mir-181,mir-320,mir-329,mir-341,mir-467,mir-7
DICER1		enzyme		0,10	4,13E-11	let-7,mir-139,mir-154,mir-181,mir-193,mir-196,mir-23,mir-29,mir-7,mir-8
Gnasas1		other		-0,90	9,76E-11	let-7,mir-186,mir-196,mir-23,mir-7,mir-8
RNA polymerase iii		complex		-0,45	1,20E-10	mir-154,mir-181,mir-506,mir-615,mir-7
cisplatin		chemical drug		-0,03	1,45E-10	let-7,mir-135,mir-140,mir-181,mir-186,mir-193,mir-196,mir-23,mir-29,mir-32,mir-467,mir-7,mir-8
IGF1R		transmembrane receptor		1,01	7,97E-10	let-7,mir-139,mir-154,mir-196,mir-299,mir-329,mir-341,mir-379,mir-467
docetaxel		chemical drug		1,08	1,57E-09	let-7,mir-181,mir-193,mir-29,mir-320,mir-7,mir-8
PARN		enzyme		1,98	3,93E-09	let-7,mir-181,mir-193,mir-379
EPHB6		kinase		1,96	1,29E-08	let-7,mir-23,mir-29,mir-32,mir-506
sirolimus		chemical drug		0,65	2,48E-08	let-7,mir-135,mir-140,mir-154,mir-181,mir-23,mir-29,mir-506,mir-8,mir-95
dalfampridine		chemical drug		-1,53	4,41E-08	let-7,mir-154,mir-23,mir-29,mir-329
BB-Cl-amidine		chemical reagent			7,53E-08	let-7,mir-23,mir-29
INSR		kinase		0,97	1,69E-07	let-7,mir-139,mir-154,mir-196,mir-299,mir-329,mir-341,mir-379,mir-467
vorinostat		chemical drug		0,53	2,85E-07	let-7,mir-140,mir-154,mir-193,mir-299,mir-379,mir-8
NF2		other		1,10	3,94E-07	let-7,mir-135,mir-196,mir-29
cocaine		chemical drug		0,81	6,21E-07	mir-154,mir-181,mir-186,mir-379,mir-467,mir-7
TNFRSF1B		transmembrane receptor		-0,97	7,13E-07	let-7,mir-320,mir-467,mir-7,mir-95
BRAF		kinase			8,80E-07	mir-135,mir-154,mir-181,mir-187,mir-193,mir-8
bicuculline		chemical - endogenous non-mammalia		-1,18	2,06E-06	mir-154,mir-23,mir-29,mir-329
DROSHA		enzyme			2,53E-06	let-7,mir-23,mir-320
PADI2		enzyme			4,93E-06	let-7,mir-23,mir-29
APC		enzyme		-0,20	6,30E-06	let-7,mir-181,mir-196,mir-379,mir-8
acetaminophen		chemical drug		-1,01	1,10E-05	let-7,mir-154,mir-193,mir-29,mir-467
VAPB		other			1,49E-05	let-7,mir-196
KDM2B		enzyme			3,07E-05	let-7,mir-181,mir-8
ANKHD1/ANKHD1-EIF4EBP3		other			3,73E-05	mir-196,mir-29
tretinoin		chemical - endogenous mammalian		0,48	4,88E-05	let-7,mir-135,mir-139,mir-154,mir-181,mir-23,mir-32,mir-329,mir-379,mir-615,mir-7
exemestane		chemical drug			6,95E-05	mir-181,mir-23
ERBB2		kinase			1,01E-04	let-7,mir-187,mir-23,mir-29,mir-320,mir-467,Mir6240,Mir6392
AGO1		translation regulator			1,36E-04	let-7,mir-8
TGFB1		growth factor		1,51	1,37E-04	let-7,mir-154,mir-181,mir-196,mir-23,mir-29,mir-320,mir-379,mir-467,mir-506,mir-8
oxaliplatin		chemical drug			1,61E-04	let-7,mir-29,mir-320
hydrogen peroxide		chemical - endogenous mammalian		-0,67	1,77E-04	let-7,mir-181,mir-193,mir-23,mir-29,mir-7
aldosterone		chemical - endogenous mammalian		0,98	1,88E-04	mir-140,mir-193,mir-23,mir-29
OIP5-AS1		other			2,24E-04	mir-154,mir-7
KHDRBS1		transcription regulator			2,59E-04	mir-29,mir-32
delta-9-tetrahydrocannabinol		chemical drug			2,96E-04	mir-139,mir-95,Mir1195
PRDM5		transcription regulator			3,65E-04	mir-135,mir-196,mir-299
6-formylindolo[3,2-b]carbazole		chemical reagent			3,76E-04	mir-29,mir-329
ILF3		transcription regulator			4,03E-04	mir-135,mir-506,mir-7
perchlorate		chemical reagent			5,14E-04	mir-135,mir-8
AGT		growth factor		1,41	5,21E-04	let-7,mir-154,mir-29,mir-329,mir-379,mir-7,mir-8
F2RL1		g-protein coupled receptor			5,65E-04	let-7,mir-23,mir-8
calcifediol		chemical - endogenous mammalian			8,54E-04	let-7,mir-8
NR0B2		ligand-dependent nuclear receptor			1,10E-03	mir-154,mir-329,mir-379
budesonide		chemical drug	-0,156	-0,79	1,44E-03	mir-139,mir-140,mir-181,mir-29

Analyses set A: hepatocyte derived transcripts

Ingenuity Canonical Pathways	-log(p-value	zScore	Ratio	Molecules
Arginine Degradation I (Arginase Pathway)	1,51		0,25	OAT
Sirtuin Signaling Pathway	4,73	-1,4142136	0,0377	ATG101,CPS1,CPT1A,MT-ND2,MT-ND3,NDUFA11,NDUFA4L2,NR1H4,PCK1,SDHC,SDHD
Ketolysis	4,28		0,3	ACAA2,ACAT2,Oxct2a/Oxct2b
Mitochondrial Dysfunction	4,23		0,0468	CPT1A,MT-CO1,MT-ND2,MT-ND3,NDUFA11,NDUFA4L2,SDHC,SDHD
Oxidative Phosphorylation	3,63	2,4494897	0,0541	MT-CO1,MT-ND2,MT-ND3,NDUFA11,SDHC,SDHD
Cancer Drug Resistance By Drug Efflux	2,97		0,069	mir-154,mir-181,mir-379,RRAS
Histidine Degradation VI	2,88		0,107	Cyp2d26,CYP7B1,HAL
LXR/RXR Activation	2,56	0	0,0407	ACACA,APOB,CYP7A1,NR1H4,S100A8
FXR/RXR Activation	2,52		0,0397	APOB,CYP7A1,G6PC1,NR1H4,VLDLR
Ketogenesis	2,5		0,182	ACAA2,ACAT2
TR/RXR Activation	2,38		0,0476	ACACA,CYP7A1,G6PC1,PCK1
Estrogen Receptor Signaling	2,34	0,4472136	0,0222	MED30,MT-ND2,MT-ND3,NDUFA11,NDUFA4L2,PGF,RRAS,SDHC,SDHD
Mevalonate Pathway I	2,29		0,143	ACAA2,ACAT2
Role of IL-17A in Psoriasis	2,29		0,143	IL17RC,S100A8
Superpathway of Citrulline Metabolism	2,23		0,133	CPS1,OAT
Glucocorticoid Receptor Signaling	2,23		0,0189	G6PC1,IL17RC,MT-ND2,MT-ND3,NDUFA11,NDUFA4L2,PC,PCK1,RRAS,SDHC,SDHD
Isoleucine Degradation I	2,07		0,111	ACAA2,ACAT2
Superpathway of Geranylgeranyldiphosphate Bi	2,07		0,111	ACAA2,ACAT2
HOTAIR Regulatory Pathway	2,05	1,3416408	0,0307	let-7,mir-29,mir-7,mir-8,SPP1
Glutaryl-CoA Degradation	1,94		0,0952	ACAA2,ACAT2
TCA Cycle II (Eukaryotic)	1,87		0,087	SDHC,SDHD
PXR/RXR Activation	1,85		0,0462	CPT1A,CYP7A1,G6PC1
LPS/IL-1 Mediated Inhibition of RXR Function	1,84		0,0236	CPT1A,Cyp2d26,CYP7A1,FMO5,GSTA5,NR1H4
Neuroprotective Role of THOP1 in Alzheimer's D	1,84	1	0,0333	GNRH1,PRSS8,SERPINA3,TMPRSS2
Pregnenolone Biosynthesis	1,73		0,0741	Cyp2d26,CYP7B1
Tryptophan Degradation III (Eukaryotic)	1,7		0,0714	ACAA2,ACAT2
Superpathway of Cholesterol Biosynthesis	1,67		0,069	ACAA2,ACAT2
Ubiquinol-10 Biosynthesis (Eukaryotic)	1,65		0,0667	Cyp2d26,CYP7B1
Biotin-carboxyl Carrier Protein Assembly	1,64		0,333	ACACA
Thyroid Cancer Signaling	1,63		0,038	CXCR4,IRS2,RRAS
Insulin Receptor Signaling	1,62	0	0,0286	GYS2,IRS2,PPP1R3D,RRAS
Chemokine Signaling	1,61		0,0375	CAMK2B,CXCR4,RRAS

Analyses set B: hepatocyte derived miRNAs

Ingenuity Canonical Pathways	-log(p-value	zScore	Ratio	Molecules
HOTAIR Regulatory Pathway	4,69	1	0,0245	let-7,mir-29,mir-7,mir-8
Cancer Drug Resistance By Drug Efflux	4,57		0,0517	mir-154,mir-181,mir-379
