# Supplementary material for: Adaptation of Oxidative Phosphorylation Machinery Compensates for Hepatic Lipotoxicity in Early Stages of MAFLD
Source: Int J Mol Sci. 2022 Jun 20;23(12):6873. doi: 10.3390/ijms23126873 (PMC9224893; doi:10.3390/ijms23126873)
Supplement: Supplementary file 1 [file ijms-23-06873-s001.zip › Ver3.-SupplementFigures_Fahlbusch et al. Adaptation of oxidative phosphorylation machinery compensates hepatic lipotoxicity in early stages of MAFLD.pdf]

SUPPLEMENTARY DATA

Adaption of oxidative phosphorylation machinery compensates hepatic lipotoxicity in early stages of MAFLD

Pia Fahlbusch[1,2], Aleksandra Nikolic[1,2], Sonja Hartwig[1,2], Sylvia Jacob[1], Ulrike Kettel[1], Cornelia Köllmer[1], Hadi Al-Hasani[1,2,3], Stefan Lehr[1,2], Dirk Müller-Wieland[4], Birgit Knebel[1,2,#,*], Jörg Kotzka[1,2,#].

[1]Institute of Clinical Biochemistry and Pathobiochemistry, German Diabetes Center at the Heinrich-Heine-University Duesseldorf, Leibniz Center for Diabetes Research; 40225 Duesseldorf, Germany;

[2]German Center for Diabetes Research (DZD), Partner Duesseldorf, 40225 Duesseldorf, Germany

[3] Medical Faculty Heinrich-Heine-University Düsseldorf, 40225 Düsseldorf Germany, 40225 Düsseldorf

[4] Clinical Research Centre, Department of Internal Medicine I, University Hospital Aachen, 52074, Aachen

[#]both authors contributed equally

***Correspondence:**
Birgit Knebel, PhD
Institute of Clinical Biochemistry and Pathobiochemistry
German Diabetes Center,
Leibniz Center for Diabetes Research
at Heinrich-Heine-University Duesseldorf
Auf'm Hennekamp 65
40225 Duesseldorf, Germany
E-mail: birgit.knebel@ddz.de
Phone: +49-211-3382 536

Supplement

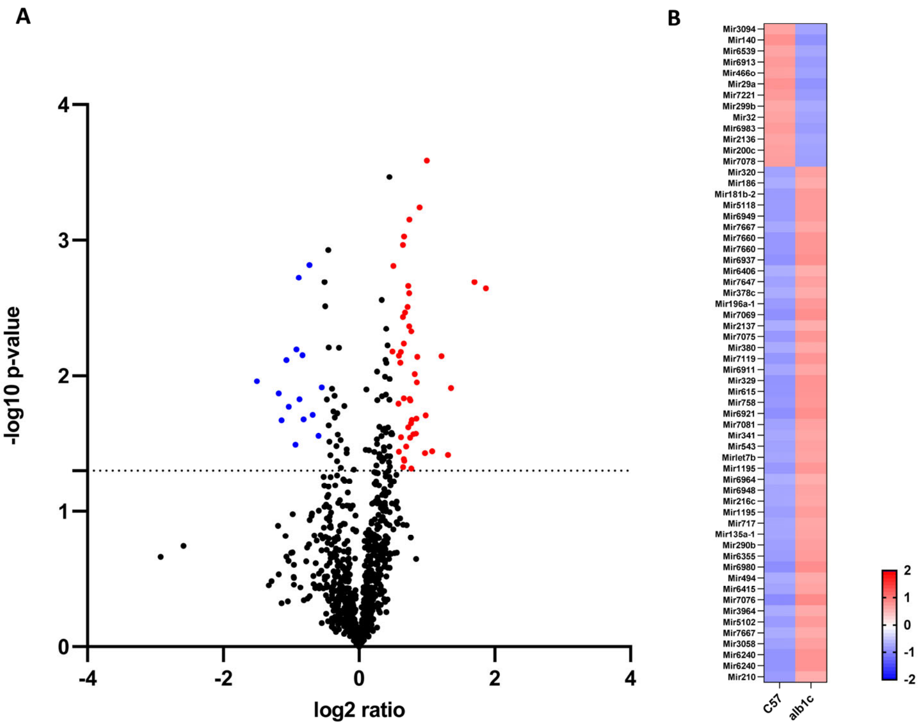

Supplementary Figure S1. **Impact of constitutive overexpression of SREBP-1c to hepatic miRNA.** (**A**) The log2 fold changes of miRNA abundance in alb-SREBP-1c vs C57Bl6 mice (n = 8 animals per condition). 1067 differentially abundant miRNA species were detected. Upregulated (red; n = 55) or downregulated proteins (blue; n = 22) were determined by Student's t-test (p > 0.05) with a 1.5 fold regulation. (**B**) Differentially regulated miRNAs. Red identifies upregulation and blue identifies downregulation of miRNAs.

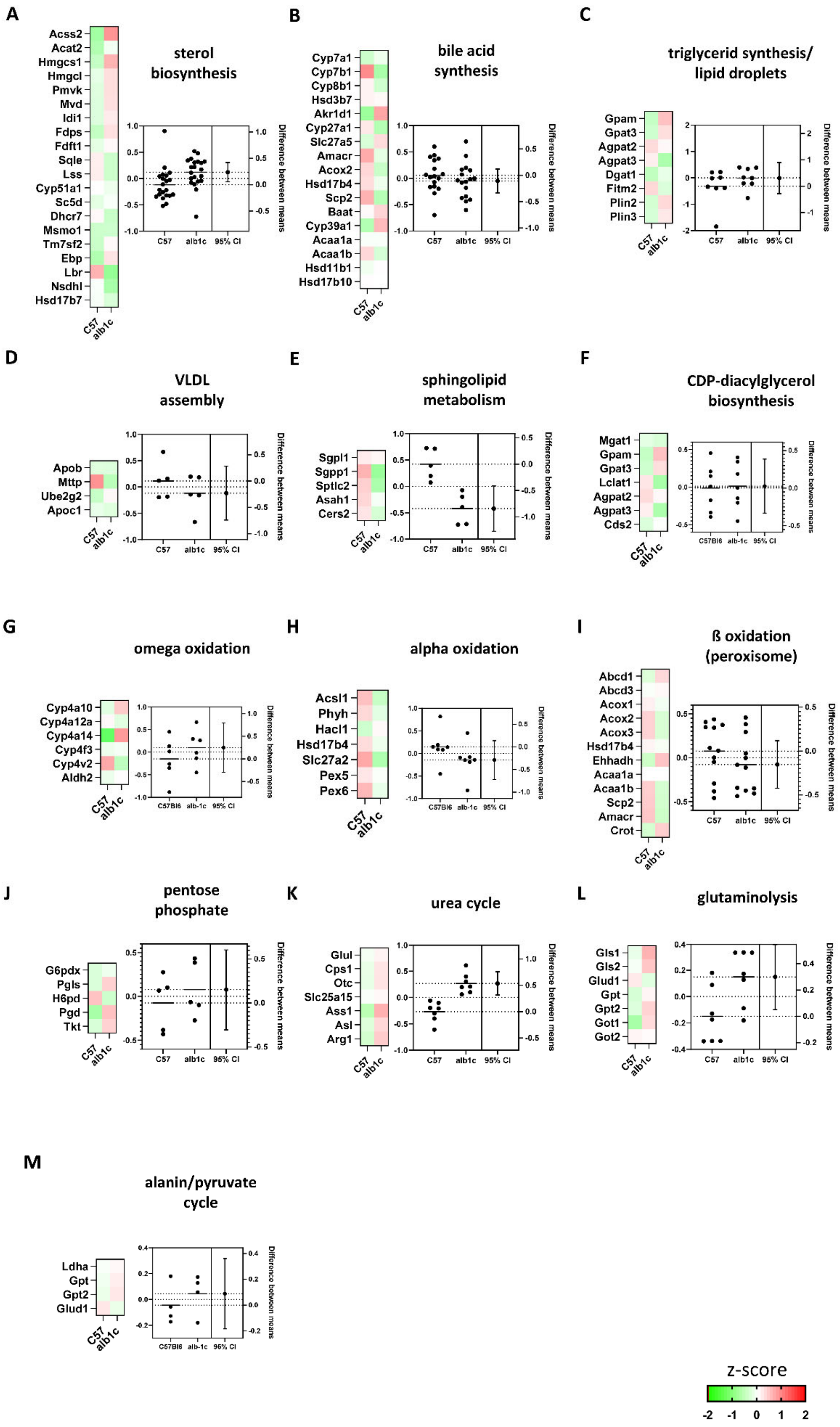
A
sterol biosynthesis
Acss2
Acat2
Hmgcs1
Hmgcl
Pmvk
Mvd
Idi1
Fdps
Fdft1
Sqle
Lss
Cyp51a1
Sc5d
Dhcr7
Msmo1
Tm7sf2
Ebp
Lbr
Nsdhl
Hsd17b7
B
bile acid synthesis
Cyp7a1
Cyp7b1
Cyp8b1
Hsd3b7
Akr1d1
Cyp27a1
Slc27a5
Amacr
Acox2
Hsd17b4
Scp2
Baat
Cyp39a1
Acaa1a
Acaa1b
Hsd11b1
Hsd17b10
C
triglycerid synthesis/ lipid droplets
Gpam
Gpat3
Agpat2
Agpat3
Dgat1
Fitm2
Plin2
Plin3
D
VLDL assembly
Apob
Mttp
Ube2g2
Apoc1
E
sphingolipid metabolism
Sgpl1
Sgpp1
Sptlc2
Asah1
Cers2
F
CDP-diacylglycerol biosynthesis
Mgat1
Gpam
Gpat3
Lclat1
Agpat2
Agpat3
Cds2
G
omega oxidation
Cyp4a10
Cyp4a12a
Cyp4a14
Cyp4f3
Cyp4v2
Aldh2
H
alpha oxidation
Acsl1
Phyh
Hacl1
Hsd17b4
Slc27a2
Pex5
Pex6
I
ß oxidation (peroxisome)
Abcd1
Abcd3
Acox1
Acox2
Acox3
Hsd17b4
Ehhadh
Acaa1a
Acaa1b
Scp2
Amacr
Crot
J
pentose phosphate
G6pdx
Pgls
H6pd
Pgd
Tkt
K
urea cycle
Glul
Cps1
Otc
Slc25a15
Ass1
Asl
Arg1
L
glutaminolysis
Gls1
Gls2
Glud1
Gpt
Gpt2
Got1
Got2
M
alanin/pyruvate cycle
Ldha
Gpt
Gpt2
Glud1
C57
alb1c
C57Bl6
alb-1c
95% CI
Difference between means
z-score
-2
-1
0
1
2

Supplementary Figure S2: Changes in biosynthesis processes in early stage MAFLD. Heat maps resulting from z-score analyses and estimation plots for each pathway are included: (**A**) Sterol biosynthesis, (**B**) bile acid synthesis, (**C**) triglyceride synthesis/ lipid droplets, (**D**) VLDL assembly, (**E**) sphingolipid metabolism, (**F**) CDP-diacylglycerol biosynthesis, (**G**) omega oxidation, (**H**) alpha oxidation, (**I**) β oxidation (peroxisome), (**J**) pentose phosphate, (**K**) urea cycle, (**L**) glutaminolysis and (**M**) alanine/pyruvate cycle. Estimation plots show differences between means of alb-SREBP-1c vs C57Bl6 (±95% CI) (mean z-score: left axis, effect size: right axis).

Supplementary Table S1: Body composition and metabolic-related parameters of C57Bl6 and alb-SREBP-1c mice. Data presented as means with 95% CI of mean. Statistical analyses were done by Wilcoxon and Mann-Whitney test.

	**C57Bl6**	**alb-SREBP-1c**	**p-value**
**N (male)**	10	10	
**Body weight (g)**	32.10 (30.28; 33.92)	35.95 (34.80; 37.08)	0.0015
**Liver weight (g)**	1.57 (1.43; 1.71)	1.93 (1.81; 2.05)	0.0005
**Liver weigth/body weight (%)**	4.92 (4.46; 5.37)	5.38 (4.95; 5.82)	0.1715
**Total fatty acid (TFA) (mg/g liver)**	25.35 (22.51; 28.19)	65.80 (55.01; 76.59)	0.0001
**TFA/liver weight (%)**	1.63 (1.42; 1.83)	3.29 (2.95; 3.83)	0.0001
**Visceral fat (g)**	0.89 (0.77; 1.02)	2.37 (2.03; 2.71)	0.0001
**Fasted blood glucose (mg/dl)**	126.8 (117.2; 136.4)	150.2 (137.8; 162.6)	0.0028
**Serum triglicerides (mg/dl)**	127.5 (106.0; 149.0)	237.4 (193.1; 281.7)	0.0001
**Serum NEFA (mg/dl)**	1.07 (0.89; 1.24)	2.38 (2.09; 2.66)	0.0001
